# Soil C/N ratio governs bacterial community assembly along an arid mountain elevational gradient

**DOI:** 10.3389/fmicb.2025.1703939

**Published:** 2026-01-20

**Authors:** Yongguang Zhang, Chaonan Li, Fanjiang Zeng, Bo Zhang, Zhihao Zhang, Chenhong Li, Hongchen Jiang

**Affiliations:** 1 Faculty of Agriculture, Forestry and Food Engineering, Yibin University, Yibin, China; 2 Xinjiang Key Laboratory of Desert Plant Roots Ecology and Vegetation Restoration, Xinjiang Institute of Ecology and Geography, Chinese Academy of Sciences, Ürümqi, China; 3 Ecological Security and Protection Key Laboratory of Sichuan Province, Mianyang Normal University, Mianyang, China; 4 School of Life Sciences, Henan University, Kaifeng, China

**Keywords:** soil ecosystem, elevational gradients, Central Kunlun Mountains, driving factors, soil organic carbon to nitrogen (C/N) ratio, ecological processes

## Abstract

The mechanisms governing soil bacterial community assembly along elevational gradients in cold-arid mountains remain poorly understood, despite their critical role in these fragile ecosystems. This study investigates these mechanisms along a pronounced climatic and vegetational gradient (1,707–3,548 m) on the northern slope of the Central Kunlun Mountains. The results show that bacterial α-diversity increased with elevation, while β-diversity exhibited a hump-shaped pattern. Mean annual precipitation (MAP) and vegetation cover were the primary drivers of these patterns, exerting stronger influences than soil pH. This suggests that arid-adapted bacteria possess unique environmental tolerances. Notably, after accounting for multicollinearity among environmental factors, the soil organic carbon-to-nitrogen (C/N) ratio emerged as the dominant factor shaping community assembly. At higher elevations (≥2,746 m), we observed increased phylogenetic clustering, linked to vegetation-driven deterministic selection via stable organic matter inputs and root exudates. However, stochastic processes still dominated the overall assembly. These findings highlight a pivotal mechanism wherein vegetation mediates bacterial community assembly primarily through modulating the soil C/N ratio in arid mountains. This study refines microbial biogeographic models by emphasizing the interplay between vegetation and soil stoichiometry under environmental stress, providing crucial insights for predicting ecosystem responses to climate change.

## 1 Introduction

Significant scientific attention has been directed towards mountain ecosystems, understood as critical biodiversity hotspots, due to their indispensable role in regulating global biogeochemical cycles and maintaining ecological stability (Wang et al., 2022; Hu et al., 2020). These environments are particularly susceptible to climate change due to pronounced environmental gradients, where temperature, precipitation, and vegetative cover exhibit substantial variation over short spatial distances (Sundqvist et al., 2013; Looby and Martin, 2020). The vital role of soil microorganisms, particularly bacteria, as the bedrock of mountain ecosystem functioning—driving nutrient cycling, decomposing organic matter, and facilitating complex plant-microbe interactions (Lehmann et al., 2017; Crowther et al., 2019). The differences in response mechanisms of bacterial diversity between arid and humid mountain ecosystems are a fundamental issue in ecology (Delgado-Baquerizo et al., 2018; Schimel, 2018). Yet, our limited understanding of the drivers behind soil bacterial diversity and community assembly across elevational gradients, especially in arid and semi-arid mountain regions, poses a significant risk to effective conservation and management strategies (Wang et al., 2022; Zhou and Ning, 2017).

Previous investigations into soil bacterial elevational diversity have yielded inconsistent patterns, including trends of decrease (Bryant et al., 2008; Nottingham et al., 2018), increase (Duan et al., 2021), hump-shaped distributions (Singh et al., 2012), or absence of clear directional trends (Delgado-Baquerizo et al., 2018). These discrepancies suggest that bacterial responses to elevation are highly context-dependent, influenced by regional climate, soil characteristics, and interspecific biotic interactions (Yang et al., 2022; Wang et al., 2023). In arid mountains, where water scarcity fundamentally shapes life, precipitation dynamics emerge not merely as a factor, but as the decisive arbiter of bacterial community composition, potentially eclipsing the influence of temperature or pH (Lai et al., 2025; Yang et al., 2021). However, the majority of existing research has focused on humid or temperate mountain systems (e.g., the Tibetan Plateau, the Andes), creating a significant knowledge gap concerning bacterial diversity in water-limited regions such as the Kunlun Mountains (Du et al., 2022; Zhang et al., 2025).

Soil pH has historically been considered a predominant factor structuring bacterial communities (Fierer and Jackson, 2006; Shen et al., 2019). Nevertheless, recent studies in arid environments challenge this long-standing paradigm, revealing that dryland bacteria exhibit a broader pH tolerance, particularly in circumneutral to alkaline soils (Zhou et al., 2024; Mod et al., 2021). For example, Zhou et al. (2024) found that arid-adapted bacterial taxa frequently flourish at pH levels exceeding 8.0, a contrast to their counterparts in acidic environments. This observation raises a critical question: does pH continue to be the primary driver of bacterial diversity in arid mountains, or do climatic factors, such as precipitation and temperature, wield a stronger control? Furthermore, while temperature generally declines with increasing elevations, precipitation patterns in mountainous regions are often more complex, frequently peaking at mid-elevations before decreasing at higher altitudes (Looby and Martin, 2020; McCain and Grytnes, 2010). This nonlinearity can foster “productivity hotspots” at intermediate elevations, where optimized moisture and temperature conditions can significantly enhance microbial activity (Wang et al., 2022). However, few studies have explicitly investigated how these climatic interactions shape bacterial communities within arid mountain contexts.

In addition to pH, soil nutrients also influence soil bacterial diversity and structure (Delgado-Baquerizo et al., 2018; Barnett et al., 2020). The ratio of soil organic carbon (SOC) to total nitrogen (TN) (C/N) often determines element cycling and nutrients availability for soil microbial communities in responses to global change (Luo et al., 2020). Soil organic matters with different C/N have various longer residence times and degradability (Bai and Cotrufo, 2022; Cotrufo et al., 2019). Numerous evidences reveal that C/N ratio significantly related to soil bacterial community diversity and composition (Wang et al., 2015; Wang et al., 2017; Delgado-Baquerizo et al., 2018; Nielsen et al., 2010; Shen et al., 2013; Shen et al., 2015; Chen et al., 2022). Vegetation is the vital source of nutrients entering into soil including provision of litter and root exudates (Prober et al., 2015; Vives-Peris et al., 2020). Plant diversity increase SOC storage by elevation carbon inputs to belowground biomass and promoting microbial necromass contribution to SOC storage (Bai and Cotrufo, 2022). In arid lands, plants exhibit inherent adaptive traits, which may lead to the selective enrichment of specific bacterial taxa (Cornwell et al., 2008; Bayranvand et al., 2021). For instance, high-elevation flora in the Kunlun Mountains produce more recalcitrant organic matter, potentially favoring the proliferation of slow-growing, oligotrophic bacteria (Pang et al., 2021). What’s more, plant functional diversity, e.g., leaf C/N ratio diversity has stronger effects on soil bacterial diversity than soil properties (Shigyo et al., 2019). However, the intricate interplay between vegetation and abiotic elements such as climate and pH makes it challenging to definitively assign primacy to either in orchestrating bacterial community development; their relative contributions are profoundly intertwined.

Modern understandings of community assembly theory propose a fundamental dialectic at play: microbial communities are simultaneously shaped by the predictable march of deterministic factors, such as environmental selection, and the unpredictable wanderings of stochastic events, like dispersal limitations and ecological drift (Zhou and Ning, 2017; Stegen et al., 2013). In harsh environments, such as arid mountains, stochasticity may assume a dominant role due to limited dispersal capacities and extreme environmental conditions (Kang et al., 2022; Ji et al., 2024). However, vegetation can introduce deterministic selection by creating spatially heterogeneous microhabitats (Huo et al., 2023; Li et al., 2018).

To address these knowledge gaps, we hypothesize that (1) Bacterial α-diversity will increase with elevation, driven by rising precipitation and vegetation cover, while β-diversity will exhibit a hump-shaped pattern due to mid-elevation peaks in moisture and productivity; (2) Climatic factors (especially precipitation) will be the dominant drivers of bacterial diversity and community composition, exerting a stronger influence than soil pH, thereby reflecting the specialized tolerances of arid-adapted bacteria; and (3) Lower C/N will enhance phylogenetic clustering at higher elevations, where stable organic matter inputs and root exudates facilitate deterministic selection, although stochastic processes will dominate overall community assembly.

Despite a wealth of research uncovering patterns of soil bacterial elevational diversity in mountain ecosystems worldwide (Shen et al., 2019; Shen et al., 2020; Duan et al., 2025; Zhu et al., 2020; Ji et al., 2020; Lai et al., 2025), the overarching narrative defining how these microbial communities are sculpted remains tantalizingly incomplete. The northern slope of the Central Kunlun Mountains presents a unique research setting, characterized by its location at the interzone between a temperate continental desert climate (extremely arid) and an alpine climate (extremely cold and arid), coupled with continuous transitional vegetation types devoid of trees over short distances. To rigorously test the hypotheses outlined above, we strategically selected the northern slope of the Central Kunlun Mountains as our research field. This cold-arid expanse, spanning an elevational gradient from 1,707 to 3,548 meters, offers a compelling setting with its dramatic climatic and vegetative shifts, perfectly poised to reveal how bacterial communities respond (Gui et al., 2010; Zhang et al., 2025). Our primary research objectives were: (1) to study how soil bacterial diversity vary along elevational gradients in arid mountains and to identify the primary environmental drivers; (2) to ascertain whether precipitation and vegetation, in tandem, override soil pH in structuring bacterial communities along arid mountain gradients; and (3) to unravel how the synergistic interplay between vegetation, climate, and soil characteristics shapes the relative dominance of deterministic versus stochastic forces orchestrating microbial community assembly. This research is not merely a regional case study, but rather a theoretical validation and extension of microbial niche construction and adaptation mechanisms in arid mountain ecosystems.

## 2 Materials and methods

### Study area and sampling design

The investigation was conducted along the northern slopes of the Central Kunlun Mountains, a region characterized by extreme aridity and pronounced elevational gradients. Seven sampling sites were strategically established between 1,707–3,548 meters above sea level to capture the complete transition from desert to alpine meadow ecosystems (Supplementary Figure S1 and Supplementary Table S1). At each elevation, we set a 15-meter square plot, containing four corner points and the intersection points of diagonals, to ensure spatial independence and account for microhabitat variability. With the above points as the intersection point of diagonals, five replicate quadrats (2 × 2 m) were designed. Within each quadrat, we collected five soil cores from the similar five points of the quadrats (0–10 cm depth) using sterilized stainless steel corers (2 cm diameter) that were flame-sterilized between samples to prevent cross-contamination, and mixed these cores evenly to form one sample. The sampling protocol was carefully timed during September 2018 to coincide with peak microbial activity at the end of the growing season. To ensure the preservation of sample fidelity, all composite samples were immediately transferred to sterile 50 mL Falcon tubes and flash-frozen in liquid nitrogen within a strict 30-min capture window. Subsequently, all samples were maintained at −80 °C to safeguard the integrity of nucleic acids for future analysis. High-precision GPS (Garmin GPSMAP 64s) was employed to record accurate geographic coordinates and elevation for each sampling location. Parallel, continuous microclimate monitoring of temperature and humidity was achieved using HOBO data loggers at each site.

### 2.1 Soil physicochemical characterization

To unlock the secrets dictating microbial community dynamics, we undertook a comprehensive physicochemical profiling of all soil samples. Following meticulous lyophilization (48 h, Labconco FreeZone) and homogenization (2 mm sieve), a suite of critical soil parameters was quantified. This included gravimetric determination of soil moisture content (Wat) (10 g subsamples, 105 °C to constant weight), pH and electrical conductivity (Con; 1:5 soil:water, equilibrated for 30 min, Mettler Toledo SevenCompact meters), total nitrogen (TN; micro-Kjeldahl digestion), soil organic carbon (SOC; potassium dichromate oxidation), available phosphorus (AP; Bray-1 extraction, colorimetric), key inorganic nitrogen forms (NH_4_^+^, NO_3_^−^; 1 M KCl extraction, continuous flow analysis), and total water-soluble salt (Sal) (the residue drying-quality method). Each measurement, conducted in triplicate with robust quality controls, provided vital insights into the drivers of microbial life.

### 2.2 Soil DNA extraction and amplification

To ensure the integrity of our molecular landscape, all analyses were meticulously shielded from external contaminants within a purpose-built clean room environment. We extracted total genomic DNA from 0.25 g soil aliquots using the PowerSoil DNA Isolation Kit (MO BIO Laboratories), refining the protocol to enhance lysis efficiency with extended bead-beating (5 min, 30 Hz) and incubation (65 °C for 10 min). Rigorous DNA quality control included agarose gel electrophoresis and NanoDrop 2000 spectrophotometry, with only samples exhibiting A260/280 ratios of 1.8–2.0 proceeding to 16S rRNA gene amplification. The V4–V5 hypervariable regions were amplified using barcoded primers 515F/909R and HotStarTaq Plus Master Mix (Qiagen) under carefully optimized conditions: initial denaturation (94 °C, 3 min); cyclic amplification (30 cycles: 94 °C, 40 s; 56 °C, 60 s; 72 °C, 60 s); and final extension (72 °C, 10 min). PCR products were closely monitored with gel electrophoresis, quantified via Qubit dsDNA HS Assay (Thermo Fisher), and pooled at equimolar concentrations. Furthermore, every batch was attended with a suite of critical extraction and PCR negative controls to sentinel for any potential contamination intrusion in what was an essential aspect of maintaining the accuracy.

### 2.3 Sequencing and bioinformatics processing

Our amplicon pipeline, engineered for precision and accuracy, commenced with library preparation and sequencing at the Beijing Genomics Institute on an Illumina NovaSeq 6000 platform employing 2 × 250 bp paired-end chemistry. The resultant raw 16S rRNA sequence data, now archived in the NCBI Sequence Read Archive (accession number PRJNA1322520), underwent a series of critical quality control checkpoints. Adapter trimming (Cutadapt v1.18) and stringent quality filtering (*Q* ≥ 30) were performed, followed by length trimming to a minimum of 200 bp within the robust QIIME2 (v2019.10) framework (Bolyen et al., 2019). Paired-end reads were then seamlessly fused (FLASH v1.2.7), requiring a minimum overlap of 20 bp and a maximum mismatch density of 0.25. Chimeric sequences, potential artifacts of amplification, were systematically purged using the UCHIME algorithm within VSEARCH (v2.14.1). Amplicon sequence variants (ASVs) were subsequently resolved via DADA2’s quality-aware error modeling, with forward reads truncated at position 240 and reverse reads at 200. Taxonomic assignment was executed against the SILVA 138 database utilizing a consensus mechanism, integrating both RDP classifier (confidence threshold 0.7) and BLASTn (*e*-value 1 ×10^−5^). Phylogenetic relationships were reconstructed using FastTree (v2.1.11) employing the GTR + CAT model and 1,000 bootstrap replicates. To ensure equitable comparison, all samples were rarefied to 7,478 high-quality sequences, establishing a standardized sequencing depth for downstream analyses (Supplementary Figure S2).

### 2.4 Community structure and functional profiling

The microeco package in R (4.3.2) was used for microbial community analyses (Liu et al., 2021). To understand the intrinsic richness and evenness of our microbial communities, we employed a multifaceted approach, examining metrics that capture both the number of distinct taxa (observed ASVs) and the distribution of individuals across those taxa (Shannon–Weaver index and analogous measures). Beta diversity was calculated using taxonomic (Bray–Curtis) distance measures. Functional potential of bacterial communities was predicted using FAPROTAX (v1.2.12) (Louca et al., 2016) with manual curation of metabolic pathways specific to arid environments. We tested the collinearity between climate, elevation and other environmental variables to reduce their autocorrelation with the function var*clus* in Hmisc package. To elucidate the mechanistic links between our environmental variables and the observed microbial community structure, we employed the distance-based redundancy analysis (dbRDA) via the “dbrda” function in the Vegan package. Subsequent partial Mantel tests, employing robust permutation testing (999 permutations), were utilized to rigorously quantify the extent to which environmental distance matrices explained the patterns of community dissimilarity.

### 2.5 Statistical analysis and modeling

Comprehensive statistical analyses were implemented in R (v4.3.2) following recent recommendations for microbial ecology studies. We applied analysis of similarity (ANOSIM, anosim function) with 999 permutations to test for significant differences among elevation groups. Partial least squares path modeling (PLSPM) was applied to evaluate direct and indirect pathways linking space, climate, vegetation, soil features and soil bacterial diversity and structure with plspm R package. To unravel the complex interplay between deterministic and stochastic forces shaping microbial community assembly, the framework proposed by Stegen et al. (2012, 2013) was used; the standardized effect size measure of the mean nearest taxon distance (ses.MNTD) and the β-nearest taxon index (βNTI) were calculated with the iCAMP R package (v1.5.3) (Ning et al., 2020). All graphical outputs were generated using ggplot2 (v3.4.2) with custom themes ensuring publication-quality visualization. Statistical significance was assessed at *α* = 0.05 unless otherwise noted, with multiple testing corrections applied where appropriate.

## 3 Results

### 3.1 Correlation between the environmental variables

With the increase of elevational gradients, environmental variables often changed simultaneously. To minimize the spatial autocorrelation, the function *varclus* was used to test the collinearity between the different environmental variables. Results indicated that mean annual precipitation (MAP) exhibited the highest similarities with mean annual temperature (MAT) and elevational gradient (Alt), followed by Wat (Supplementary Table S2). While SOC exhibited the highest spearman *ρ*^2^ (0.93) with TN. The similarities between other environmental variables were <0.9. For arid-mountains, MAP played vital roles in ecosystems; SOC was a resource available for soil bacteria. Thus, to reveal the real variables in driving soil bacterial community, MAT, Alt, Wat and TN were removed for the latter analysis.

### 3.2 Soil bacterial community composition and diversity along elevational gradients

**Figure 1:**
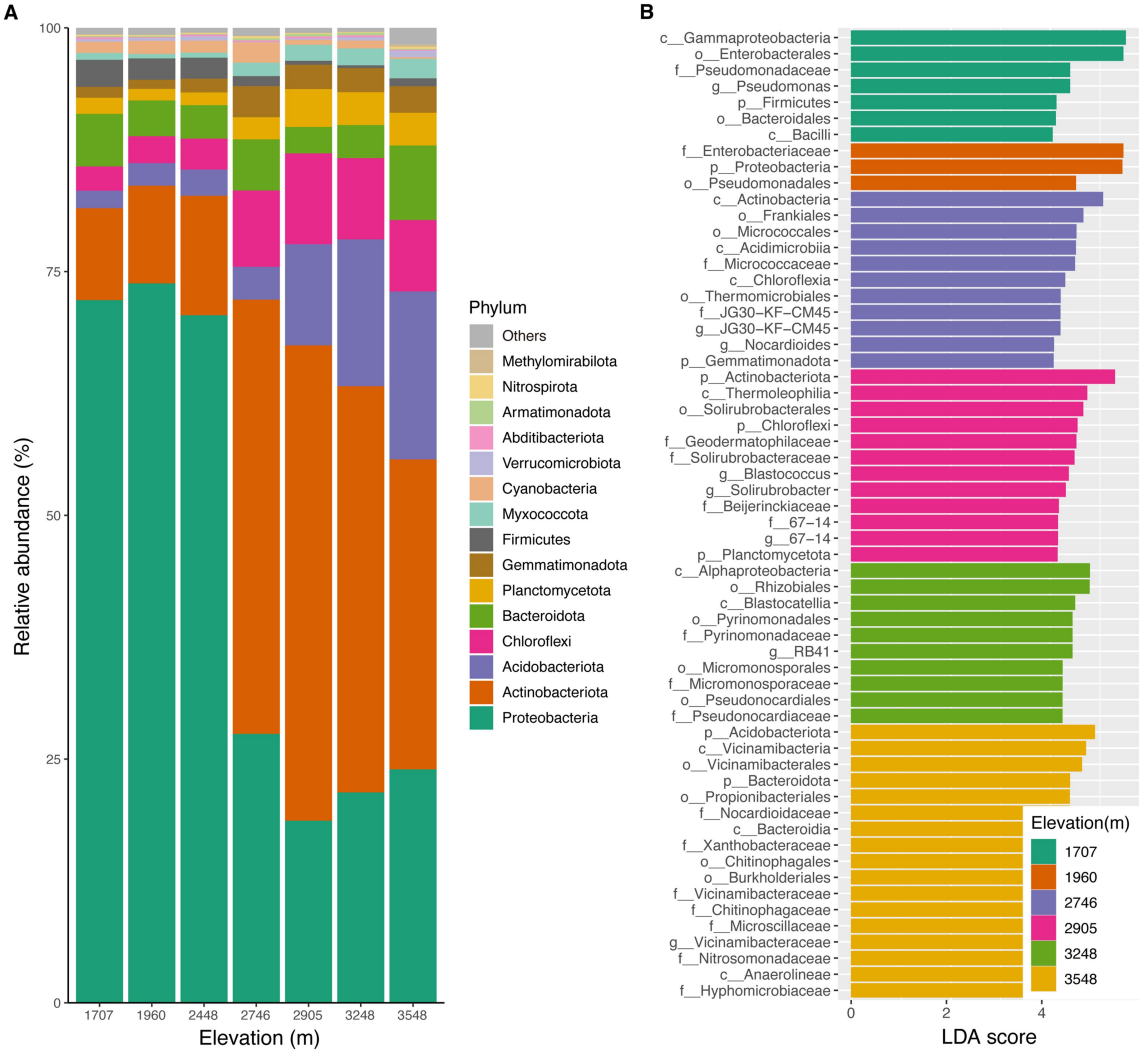
Composition of soil bacterial community at the phylum level. **(A)** Relative abundances of the dominant bacterial phyla in the soil bacterial community. **(B)** Different biomarkers among the different altitudes based on linear discriminant analysis (LDA).

The analysis of soil samples revealed a remarkably diverse microbial community, with over a million quality-filtered sequences contributing to the identification of more than 4,000 unique ASVs. These ASVs, characterized against the Silva 138 database, represent a substantial snapshot of the soil’s microbial richness. Sequences were classified into bacterial and archaeal domains, with 94.45 and 5.55% of reads, respectively. At the phylum level, soil bacterial community comprised 31 phyla and other unidentified sequences across all elevational sites. The dominant phyla were *Proteobacteria* (42.5% relative abundance) and *Actinobacteria* (26.0%). Additionally, *Acidobacteriota*, *Chloroflexi*, *Bacteroidota* and *Planctomycetota* consistently represented more than 2.0% of the relative abundance (Figure 1A). The relative abundance of various phyla exhibited distinct trends along the elevational gradient, including increasing, hump-shaped, and decreasing patterns (Supplementary Figure S3). Similar altitudinal shifts were also observed at the class level (Supplementary Figure S4), indicating that elevational diversity patterns of soil bacteria are taxon-specific.

To explore potential ecological biomarkers within soil bacterial communities, we applied LDA analysis, revealing candidate signatures (LDA >4, *p* < 0.05) at each of the seven elevations (Figure 1B). Notably, the 3,548 m site yielded the largest set of these candidate indicators (17). These findings suggest potential elevational gradients influencing community composition.

**Figure 2:**
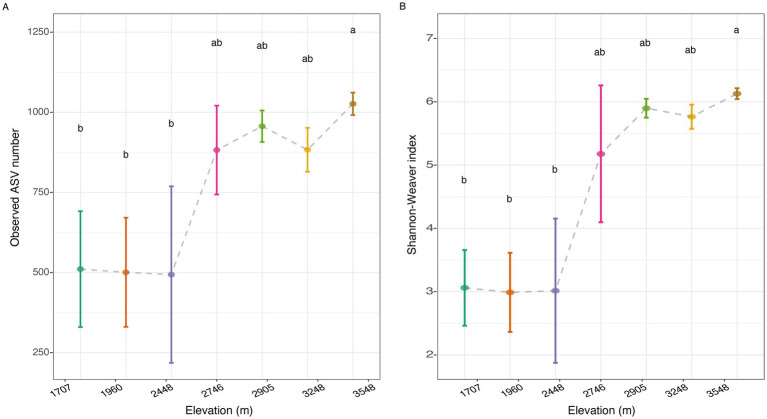
Shifts in observed ASV number **(A)** and Shannon-Weaver index **(B)** of soil bacterial community across the elevational gradients.

While our prior work (Zhang et al., 2025) extensively characterized environmental variables across the elevational gradient, Spearman’s correlation analysis surprisingly failed to establish significant associations between the relative abundance of the top 15 bacterial phyla and environmental factors (Supplementary Figure S5). Kruskal–Wallis_dunn’s test indicated a significant increasing trend in observed ASV number (Figure 2A) and Shannon-Weaver index (Figure 2B) across elevational gradients, with the highest values recorded at 3,548 m. However, the α-diversity indices at the lower elevations (1,707 m, 1,960 m, and 2,448 m) did not exhibit pronounced changes (Figure 2). Spearman’s correlation analysis further highlighted MAP as the most strongly correlated environmental factor with α-diversity indices (Supplementary Figure S6 and Supplementary Table S3). In addition, pH, Con and Sal also demonstrated significant correlative relationships with α-diversity metrics, while not AP, NO_3_^−^ and NH_4_^+^ (Supplementary Table S3).

**Figure 3:**
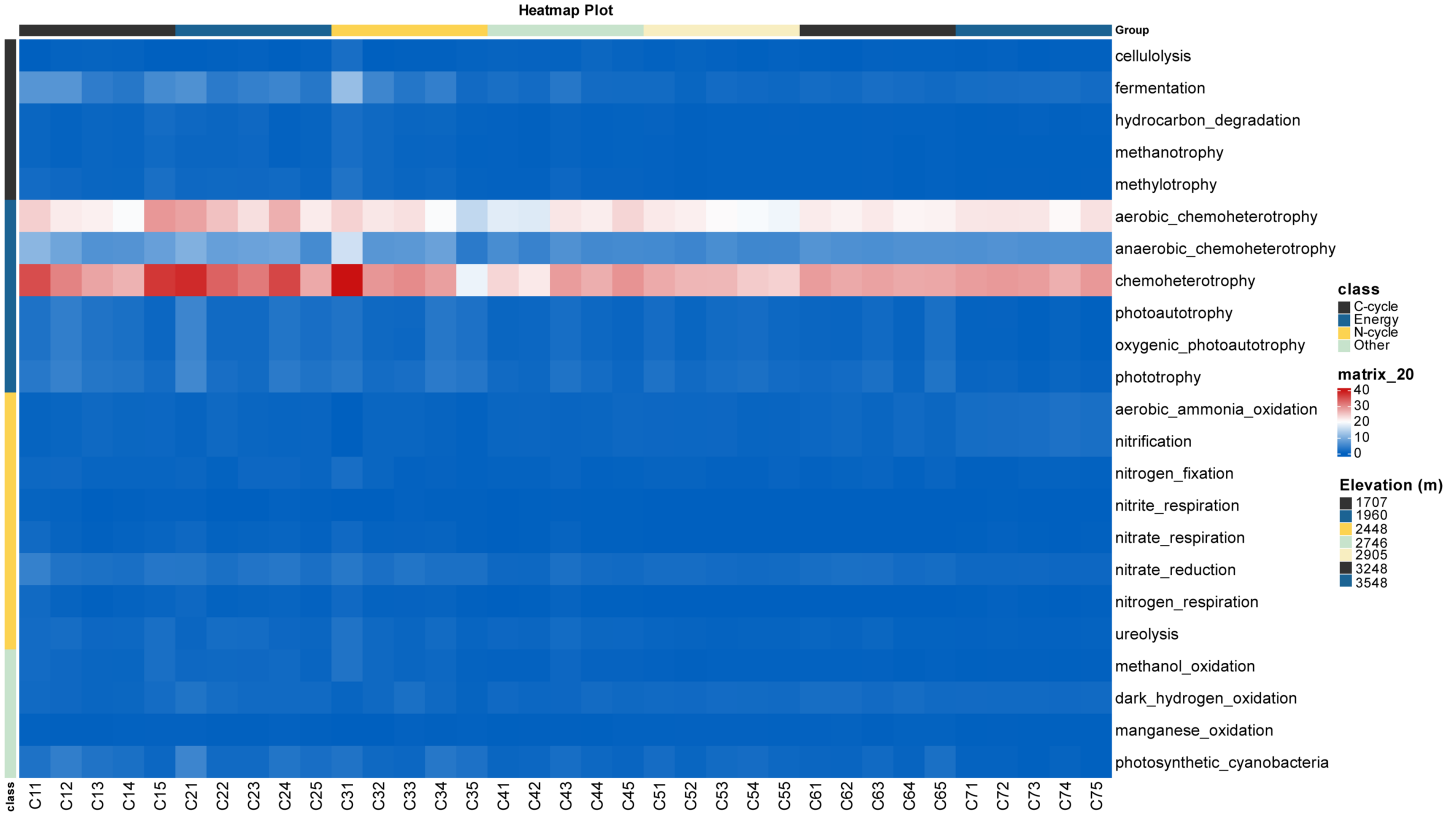
The significant correlation of environmental variables and the abundances of the predicted functional groups.

To investigate the presence of known biogeochemical cycling functions within our soil bacterial community, we conducted an annotation process using the FAPROTAX database (v1.2.12). The analysis indicated that the number of predicted functional genes generally decreased with increasing elevational gradients, such as the profiles of chemoheterotrophy and other 22 functional genes (Figure 3). The relative abundances of these predicted functional genes also varied along the altitudinal transect (Supplementary Figure S7). Among the five major categories of predicted functional genes, those involved in energy yield, such as chemoheterophy (aerobic_chemoheterotrophy and anaerobic_chemoheterotrophy) and phototrophy (photoautotrophy and photoheterotrophy) were the most abundant, followed by nitrogen-related genes (N_genes), carbon-related genes (C_genes), and other functional categories (Supplementary Figure S8). Spearman’s correlation analysis revealed significant correlations between MAP, pH and Veg with the abundances of predicted functional genes (Supplementary Figure S9). In addition, some soil characteristics, such as SOC, Sal and Con also exhibited significant correlation with most predicted functional genes except for NO_3_^−^, NH_4_^+^ and AP. These findings suggest that the elevational distribution patterns of bacterial taxa influence their ecological functions.

### 3.3 Shifts in soil bacterial community structure along elevational gradients

**Figure 4:**
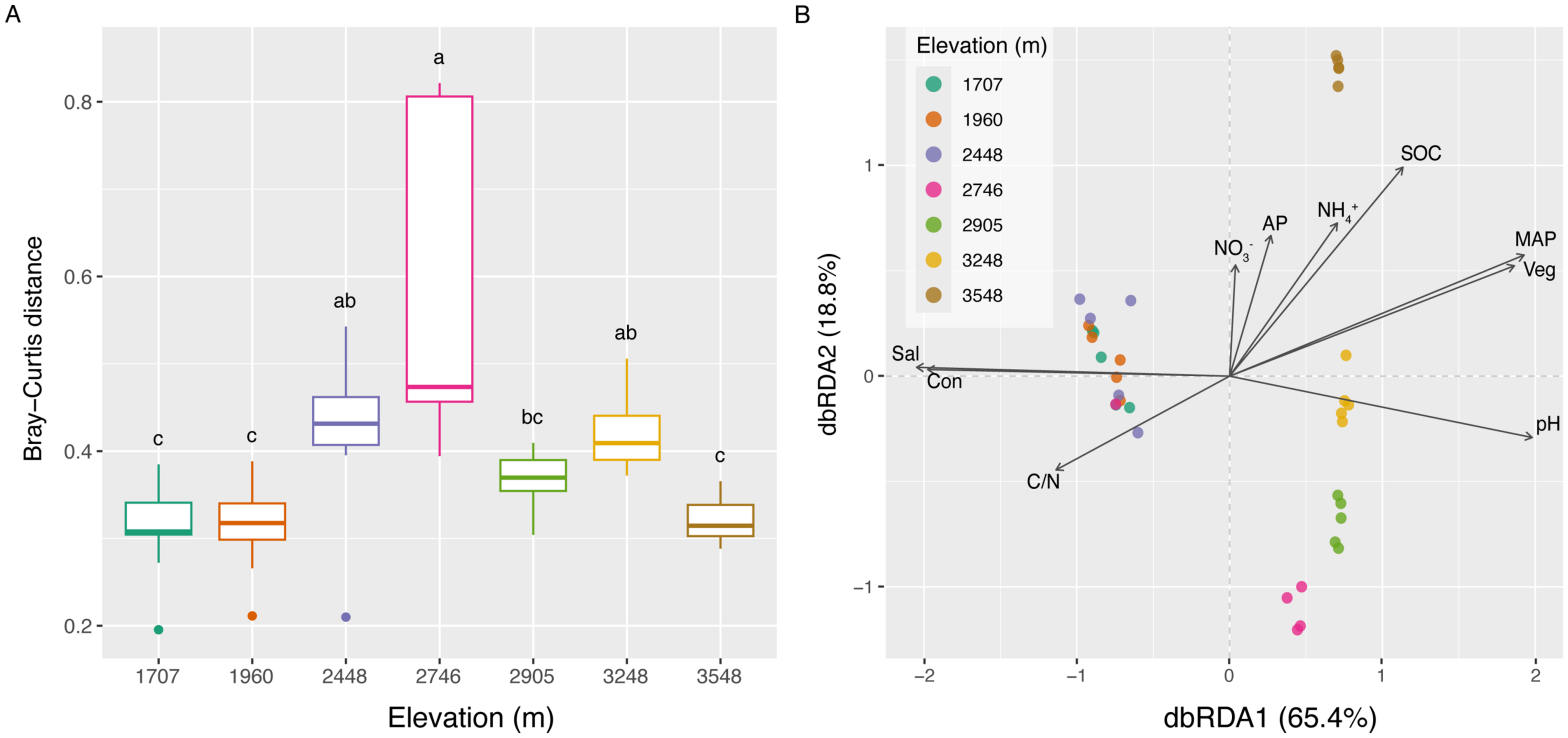
Shifts in the composition of soil bacterial community along the elevational gradients and their relationships with the environmental factors. **(A)** Differences of the Bray–Curtis distance. **(B)** Effects of the environmental factors on the composition of soil bacterial community based on dbRDA analysis. AP, available phosphorus; C/N, the ratio of soil organic carbon to total nitrogen; Con, electrical conductivity; MAP, mean annual precipitation; NH_4_^+^, ammonium nitrogen; NO_3_^−^, nitrate nitrogen; Sal, total water-soluble salt; SOC, soil organic carbon; Veg, vegetation cover.

A striking pattern of elevational zonation was evident in the soil bacterial communities. ANOSIM analysis, using Bray–Curtis distances, verified significant differences in community structure across elevational gradients (*p* < 0.05, Supplementary Table S4). β-diversity exhibited a clear hump-shaped trend with increasing elevation (Figure 4A). dbRDA further illuminated this pattern, revealing significant correlations between community structure and the measured environmental variables. Bacterial communities at lower elevations (1,707 m, 1,960 m, and 2,448 m) showed a degree of similarity, clustering together, while those at higher elevations were distinct (Figure 4B). The dominant environmental variables shaping this community structure included MAP, Veg, and pH, alongside other factors such as SOC, Sal and C/N.

**Figure 5:**
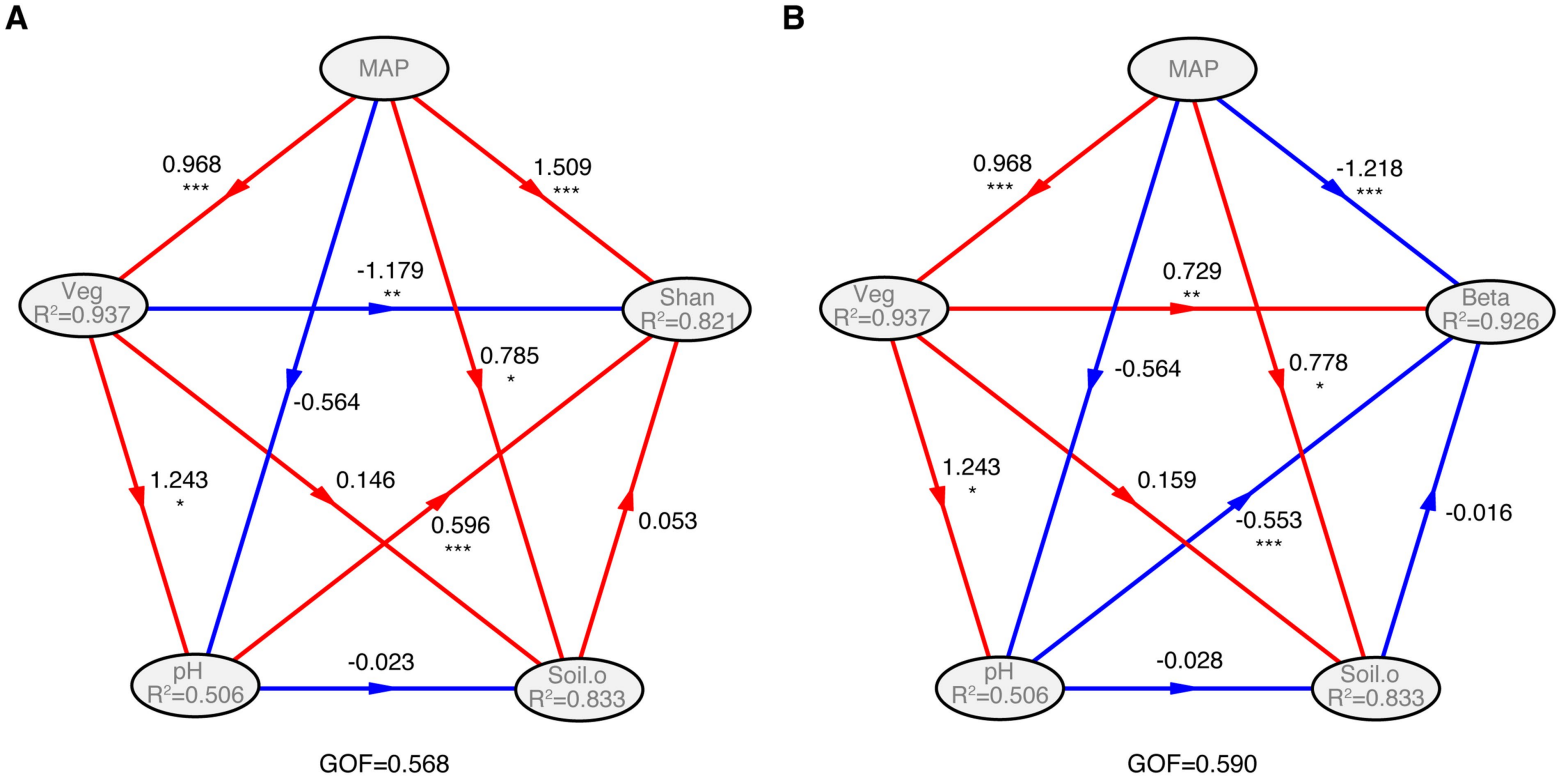
PLSPM analysis the relation of environmental variables with α-diversity (Shannon–Weaver index) **(A)** and the structure of soil bacterial community **(B)**. The models were constructed at the conditions of the removal of four variables (elevation, mean annual temperature, total nitrogen, and soil moisture content), which showed higher collinearities than 0.9 (Spearman *ρ*^2^ > 0.9) calculated with the function varclus in the Hmisc package. MAP, mean annual precipitation; Soil.o includes: AP, available phosphorus; C/N, the ratio of soil organic carbon to total nitrogen; Con, electronic conductivity; NH_4_^+^, ammonium nitrogen; NO_3_^−^, nitrate nitrogen; Sal, total water-soluble salt; SOC, soil organic carbon. Veg, vegetation coverage. Shan, Shannon–Weaver index; Beta, the first two axes of PCoA; GOF, goodness of fit. Significance level, ^*^p < 0.05, ^**^p < 0.01, and ^***^p < 0.001.

At lower elevations (1,707 m, 1,960 m, and 2,448 m), soil bacterial communities exhibited a limited correlation with environmental factors, with only MAP and Veg showing significant associations with community structure. This contrasts sharply with higher elevations, where six distinct environmental factors, alongside MAP, pH, SOC, Con, Sal and NO_3_^−^, collectively exerted a strong deterministic influence (Table 1), indicating that niche-based assembly mechanisms become increasingly important at greater altitudes and that broad environmental structuring is primarily a high-elevation phenomenon. Further PLSPM analysis indicated that MAP is the most influential driver of species diversity (Shannon–Weaver index) and bacterial community structure, followed by Veg and pH (Figure 5). Additionally, SOC, Sal, Con, C/N and geological distance (Geo) also influenced the elevational diversity patterns of the bacterial community (Table 1).

**Table 1:** Partial Mantel analysis the effects of environmental variables and geological features on the composition of soil bacterial communities.

Variables	All elevational gradients		Lower elevational gradients		Higher elevational gradients	
	*r*	*p*-values	*r*	*p*-values	*r*	*p*-values
pH	**0.479**	0.001	−0.126	0.737	**0.453**	0.001
Con	**0.365**	0.001	0.041	0.361	**0.578**	0.001
Sal	**0.426**	0.001	0.125	0.147	**0.428**	0.001
AP	−0.078	0.992	0.012	0.461	−0.081	0.855
NO_3_^−^	−0.210	1.000	−0.203	0.921	**0.325**	0.001
NH_4_^+^	−0.009	0.554	−0.009	0.653	0.221	0.054
Veg	−0.124	0.997	**0.280**	0.033	0.040	0.371
C/N	**0.091**	0.034	−0.244	0.990	0.089	0.225
SOC	**0.147**	0.003	−0.124	0.787	**0.606**	0.001
MAP	**0.716**	0.001	**0.287**	0.026	**0.399**	0.001
Geo (controlling all environmental variables)	**0.242**	0.001	−0.111	0.699	**0.486**	0.001
Env (controlling geological distance)	**0.546**	0.001	−0.028	0.517	**0.709**	0.001

AP, available phosphorus; C/N, the ratio of soil organic carbon to total nitrogen; Con, electronic conductivity; MAP, mean annual precipitation; NH_4_^+^, ammonium nitrogen; NO_3_^−^, nitrate nitrogen; Sal, total water-soluble salt; SOC, soil organic carbon; Veg, vegetation cover; Geo, geological distance; Env, containing the 10 variables listed. The bold values mean that they are significant (*p* < 0.05) among the tested variables.

### 3.4 Ecological processes shaping soil bacterial community across elevational gradients

**Figure 6:**
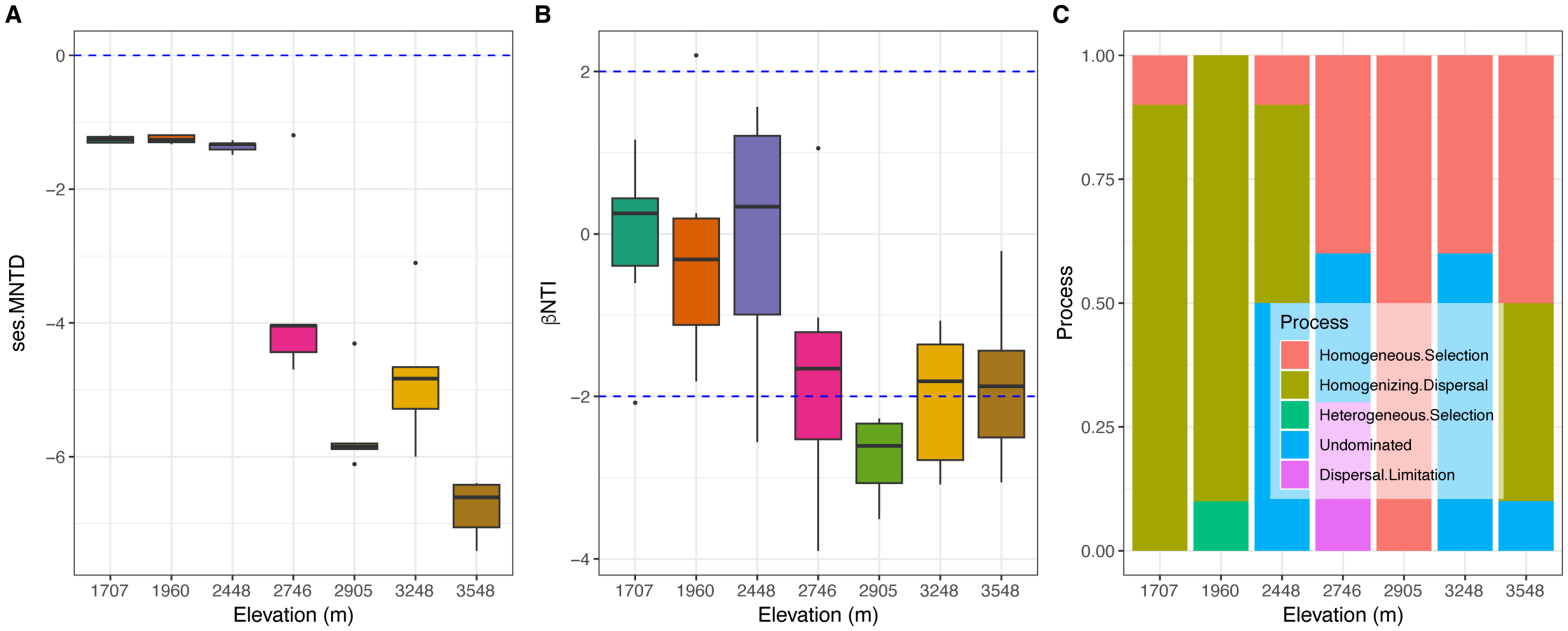
Profiles of phylogenetic diversities and the ecological processes of soil bacterial community along the elevational gradients. **(A)** Differences of the values of ses.MNTD. **(B)** Profiles of the values of βNTI. **(C)** Effects of ecological processes on the soil bacterial community construction.

Phylogenetic null model analysis demonstrated that observed pairwise dissimilarities in soil bacterial communities (ses.MNTD) were consistently less dispersed than expected by chance, particularly at higher elevations where this effect intensified (median values <0, decreasing with altitude). This pattern strongly suggests that rigorous environmental filtering at higher altitudes is imposing common traits, leading to increasingly phylogenetically conserved bacterial communities (Figure 6A). The βNTI values were divided into two distinct groups: values around zero (≤2,448 m) and values around −2 (≥2,746 m), both decreasing with increasing elevation (Figure 6B). These results suggest a transition in soil bacterial community assembly, where stochasticity is the prevalent force across a range of conditions. Notably, at higher elevations, this random element recedes, yielding dominance to selective pressures that dictate community membership (Figure 6C). Partial Mantel tests revealed that C/N was the foremost agent controlling community membership, highlighting its profound impact (Table 2). In addition, Con, Sal, NO_3_^−^, and Veg significantly aligned with community structure, underscoring the deterministic nature of environmental control. Furthermore, linear regression analyses confirmed the significance of these variables, with the exception of pH, NH_4_^+^, SOC and MAP (Table 3).

**Table 2:** Partial Mantel tests between environmental variables and β-nearest taxon index (βNTI) based on the Spearman rank correlation.

Variables	All elevational gradients		Lower elevational gradients		Higher elevational gradients	
	*R* ^2^	*p*-values	*R* ^2^	*p*-values	*R* ^2^	*p*-values
pH	−0.059	0.872	0.090	0.284	−0.142	0.97
Con	**0.231**	0.002	0.015	0.424	**0.152**	0.0089
Sal	**0.191**	0.006	−0.033	0.601	**0.273**	0.004
AP	0.022	0.352	0.119	0.204	−0.181	0.983
NO_3_^−^	**0.223**	0.001	−0.041	0.595	−0.186	0.989
NH_4_^+^	0.049	0.295	0.007	0.414	−0.027	0.628
Veg	**0.110**	0.031	−0.131	0.945	**0.360**	0.001
C/N	**0.265**	0.001	−0.021	0.535	**0.183**	0.039
SOC	0.070	0.212	−0.170	0.934	−0.266	0.999
MAP	0.087	0.082	−0.131	0.937	0.057	0.177
Env	0.1045	0.05	−0.04153	0.632	0.05256	0.2

AP, available phosphorus; C/N, the ratio of soil organic carbon to total nitrogen; Con, electronic conductivity; MAP, mean annual precipitation; NH_4_^+^, ammonium nitrogen; NO_3_^−^, nitrate nitrogen; Sal, total water-soluble salt; SOC, soil organic carbon. Veg, vegetation cover; Env, containing the 10 variables listed. The bold values mean that they are significant (*p* < 0.05) among the tested variables.

**Table 3:** Linear regression analysis of the effect of the environmental variables on β-nearest taxon index (βNTI) of soil bacterial community.

Variables	All elevational gradients		Lower elevational gradients		Higher elevational gradients	
	*R* ^2^	*p*-value	*R* ^2^	*p*-value	*R* ^2^	*p*-value
pH	**<0.01**	0.032	0.02	0.201	<0.01	0.73
Con	**0.04**	0.001	<0.01	0.357	**0.04**	0.005
Sal	**0.01**	0.004	<0.01	0.546	**0.05**	0.001
AP	<0.01	0.311	<0.01	0.313	0.02	0.059
NO_3_^−^	**0.06**	<0.001	<0.01	0.901	<0.01	0.605
NH_4_^+^	**0.01**	0.006	<0.01	0.317	<0.01	0.249
Veg	**0.01**	0.01	<0.01	0.534	**0.1**	<0.001
C/N	**0.07**	<0.001	<0.01	0.394	**0.05**	0.003
SOC	**0.02**	0.002	**0.04**	0.038	**0.07**	<0.001
MAP	**0.01**	0.008	<0.01	0.521	**0.03**	0.02
Env	**0.02**	0.001	<0.01	0.854	**0.03**	0.025

AP, available phosphorus; C/N, the ratio of soil organic carbon to total nitrogen; Con, electronic conductivity; MAP, mean annual precipitation; NH_4_^+^, ammonium nitrogen; NO_3_^−^, nitrate nitrogen; Sal, total water-soluble salt; SOC, soil organic carbon. Veg, vegetation cover. Env, containing the 10 variables listed. The bold values mean that they are significant (*p* < 0.05) among the tested variables.

## 4 Discussion

### 4.1 Soil bacterial diversity and community composition, and their predictors across elevational gradients

Elevational diversity patterns in soil bacterial communities have been extensively studied, revealing varied trends such as decrease, increase, unimodal (one-humped), U-shaped, and no significant trends with increasing altitude (Wang et al., 2022; Looby and Martin, 2020; Nottingham et al., 2018; Delgado-Baquerizo et al., 2018). An increasing trend in species diversity with elevation, as observed in this study, is relatively uncommon (McCain and Grytnes, 2010). The observed trend is likely orchestrated by an overarching increase in climatic favorability towards higher altitudes. Specifically, ascending through this mountain ecosystem, we observed a notable uplift in precipitation (Looby and Martin, 2020). This climatic shift, from 1,707 to 3,548 m over an 1,800 m elevational span, coincided with a robust expansion of soil bacterial alpha-diversity in our study (Figure 2). Different bacterial taxa exhibited distinct responses in their relative abundances with increasing altitude (Figure 1; Supplementary Figures S3, S4). For instance, the dominant phylum, *Proteobacteria*, showed a decreasing trend, while *Actinobacteria* displayed a unimodal pattern and *Acidobacteriota* increased (Supplementary Figure S3). These differential responses are likely linked to the unique physiological characteristics of each taxon in adapting to the environmental shifts associated with increasing elevation. This inference is partially aligned with the precipitation hypothesis of elevational diversity patterns, as advanced by McCain and Grytnes (2010), due to the observed significant correlations between a range of environmental variables and the relative abundances of various phyla, along with α-diversity indices (Supplementary Figures S5, S6).

Further PLSPM analysis demonstrated that MAP over Veg and pH, significantly influenced soil bacterial α-diversity (Shannon index) (Figure 5A). Mountain ecosystems, especially in arid regions, often rely on elevational gradients for precipitation generation (Viviroli and Weingartner, 2004). Precipitation directly influenced vegetation characteristics, e.g., Veg, which also exhibited strong correlations with bacterial α-diversity (Supplementary Figure S6). For example, the dominant phyla, *Proteobacteria* and *Actinobacteria*, showed significantly positive correlations with MAP and Veg, respectively. Environmental filtering is a known mechanism by which specific soil bacterial taxa become established in habitats characterized by particular climatic conditions, soil pH levels, and dominant vegetation types (Delgado-Baquerizo et al., 2018; Karimi et al., 2018). These filters effectively select for bacterial communities adapted to those prevailing environmental parameters. While aligning with the established trends documented by Duan et al. (2021), our work diverges from the observations of Nottingham et al. (2018), Shen et al. (2020), and Singh et al. (2012). These discrepancies might stem from the harsher climatic conditions, particularly lower MAP, experienced on the north slope of the Central Kunlun Mountains compared to the transects investigated in those earlier studies (Pang et al., 2021).

The soil bacterial communities along elevational gradients reveal distinct ecological signatures, with their varied compositions serving as markers of underlying environmental and spatial heterogeneity. Our study revealed a unimodal trend in soil bacterial community composition with increasing altitude, with the highest Bray–Curtis distance observed at 2,746 m (Figure 4A). Partial Mantel test and PLSPM analysis revealed that MAP exerted a striking influence on bacterial community structure (Table 1 and Figure 5B). This is consistent with the observation that precipitation is often a critical factor structuring soil bacterial communities in arid and semiarid ecosystems (Maestre et al., 2015). For instance, Angel et al. (2010) found that precipitation gradients and vegetation cover were the main drivers of soil bacterial community variation along a steep precipitation gradient. In our study, the MAP at 2,746 m (138.8 mm) falls within a precipitation threshold range (typically 91–166 mm) known to significantly influence soil bacterial communities (Lai et al., 2025). Furthermore, the impact of environmental variables on soil bacterial community composition often varies across elevational gradients. The mid-elevation site at 2,746 m may represent a turning point. At lower altitudes (<2,746 m), only MAP and Veg significantly correlated with bacterial community composition, whereas seven variables were significantly correlated at higher altitudes (≥2,746 m) (Table 1). Consistent with the elevational climate model (ECM) proposed by McCain and Grytnes (2010), Looby and Martin (2020), our findings suggest that the warmest-wettest conditions likely occurred at intermediate elevations on the north slope of the Central Kunlun Mountains.

Veg also played a significant role, acting as a key intermediary that strongly structured the soil bacterial community composition through its influence on soil characteristics (Figures 4B, 5B). This is likely because plant productivity, which peaks at mid-elevations, leads to increased resource availability (McCain and Grytnes, 2010). Functional traits of plant communities, such as diversity, density, biomass, and C/N, can profoundly influence soil bacterial communities by delivering nutrients to the soil (Shigyo et al., 2019; Prober et al., 2015). These results support our first hypothesis that the unimodal pattern of soil bacterial β-diversity is primarily driven by mid-elevation peaks in moisture and productivity.

Additionally, pH and other variables also significantly affected soil bacterial community composition (Table 1 and Figures 4B, 5B). The relative abundances of soil bacterial predicted functional groups exhibited regular changes along the elevational gradient (Supplementary Figures S7, S8) and were significantly correlated with MAP, Veg, pH and other variables (Supplementary Figure S9). These findings underscore how elevational distribution patterns of species richness within different bacterial taxa influence their ecological functions.

### 4.2 Climate dominate soil bacterial α-diversity and composition with stronger effects than soil pH

While a substantial body of prior research, spanning several decades, has underscored soil pH as a major determinant of bacterial diversity across elevational gradients (Shen et al., 2013; Shen et al., 2019; Shen et al., 2020; Delgado-Baquerizo et al., 2018; Tian et al., 2021), our findings present a more nuanced picture. Despite a statistically significant association between soil pH and bacterial α-diversity and community composition (Supplementary Table S3 and Figure 5A; Supplementary Figure S6) in this study, the pervasive influence of MAP—emerged as a far more potent force (Figure 5). Several factors may explain this observation: (1) Soil bacteria in arid mountain environments may have evolved adaptive strategies to survive harsh conditions, such as enhanced tolerance to alkaline conditions and exhibiting alkaline optima (Zhou et al., 2024). A prior investigation into the local dryland environment suggested that variations in soil pH did not significantly correlate with levels of bacterial α-diversity (Fierer and Jackson, 2006). (2) Vegetation can modify soil pH through root exudates, which contain organic compounds that can alter rhizosphere pH by releasing OH^−^ and HCO_3_^−^ (Dakora and Phillips, 2002). Additionally, plant residue decomposition can influence soil pH through processes like decarboxylation of organic anions, ammonification, and subsequent nitrification (Xu et al., 2006). Within this study, soil pH values, typically between 6.9 and 8.0, indicated a generally neutral to slightly alkaline environment, a range that, while not extreme, may have nonetheless subtly influenced bacterial assemblages. Consequently, while climate variables (especially MAP) demonstrably drove α-diversity and community structure more forcefully, they did so within the context of this relatively stable pH, likely interacting with the inherent arid-adapted tolerances of the local bacterial communities.

### 4.3 Soil C/N mainly shapes soil bacterial community assembly across elevational gradients

Soil bacterial communities often display increased phylogenetic clustering with increasing altitude (Wang et al., 2012; Cho et al., 2019; Wan et al., 2021; Zhang et al., 2018). Phylogenetic clustering is commonly observed when environmental filters at a local scale strongly favor species possessing similar traits, which are often shared by closely related lineages. This implies that species unable to align with these prevalent local conditions, regardless of their phylogenetic relatedness to the existing community, are less likely to persist (Li et al., 2015). Evidence points to habitat filtering and biotic competition playing significant roles in the phylogenetic clustering of soil bacterial communities (Horner-Devine and Bohannan, 2006, Goberna et al., 2014), especially under extreme pH conditions (Tripathi et al., 2018) and limited carbon resources available (Zhang et al., 2018). In our study, observed ses.MNTD values were consistently below zero and decreased with increasing elevational gradients, indicating a trend of increasing phylogenetic clustering from lower to higher altitudes (Figure 6A). This points to a less diverse incoming pool or a more restrictive environment at higher elevations, leading to soil bacterial communities composed of closely related lineages, in contrast to the broader phylogenetic spectrum observed at lower elevations. This phenomenon may be attributable to the increased resources availability, which is mainly derived from aboveground vegetation (Gui et al., 2010; Zhang et al., 2025) at higher altitudes. These factors positively influence soil nutrients, potentially leading to increased bacterial richness but favoring communities composed of more closely related bacteria (Goberna et al., 2016).

It is widely accepted that both stochastic and deterministic processes influence bacterial community assembly (Wang et al., 2013; Zhou and Ning, 2017; Stegen et al., 2013; Stegen et al., 2012). However, the balance between these processes can vary depending on environmental factors, temporal, and spatial scales (Ning et al., 2020; Wang et al., 2022). Across the entire elevational transect, stochastic processes, characterized by homogenizing dispersal and undominated processes, appeared to be the dominant drivers of soil bacterial community assembly (Figure 6C). This aligns with previous studies reporting stochastic dominance along elevational gradients (Ji et al., 2024; Kang et al., 2022), although the specific ecological processes identified, such as homogenizing dispersal, differed. This variation may be attributable to ecosystem-specific conditions. Consistent with the shifts in ses.MNTD, βNTI values could be categorized into two groups: lower elevational gradients and higher elevational gradients (Figure 6B). While lower elevations showed a stronger signal of stochasticity, particularly from homogenizing dispersal, deterministic factors were not entirely absent in shaping community assembly. Conversely, at higher elevations, deterministic processes like homogeneous selection appeared to exert a more pronounced influence. According to Stegen et al. (2015), homogenizing dispersal dominates in weakly selective environments with high dispersal rates, while homogeneous selection prevails in strongly selective and uniform environments. The transition between these ecological processes supports the notion that the comprehensive soil environments at higher elevational sites were significantly different from those at lower elevations (Zhang et al., 2025), evidenced by factors like higher precipitation and Veg.

Environmental variables significantly influence the phylogenetic structure of soil bacterial assemblages, as evidenced by robust partial Mantel and linear regression analyses (Tables 2, 3). C/N demonstrated the strongest positive association with the null model in determining the extent of phylogenetic conservatism (βNTI = 0.265, *p* = 0.001), indicating its critical role in structuring soil bacterial communities. This finding diverges from previous studies that emphasized soil pH, mean annual precipitation (MAP), and temperature as primary drivers of bacterial community assembly (Cho et al., 2019; Nottingham et al., 2018; Yang et al., 2022; Shen et al., 2019; Ni et al., 2021; Tripathi et al., 2018). The discrepancy may stem from the fundamental importance of C/N availability in nutrient provisioning. Evidences have shown that soil C/N not only affects bacterial diversity and biogeographic distribution (Shen et al., 2015; Shen et al., 2013; Nielsen et al., 2010; Chen et al., 2022; Delgado-Baquerizo et al., 2018), but also relates to bacterial community assembly (Kang et al., 2023). At higher altitudes, a reduced soil C/N ratio, despite increasing SOC and TN content, implies less readily accessible organic carbon for the soil bacterial community, leading to intensified competition for carbon resources. Our data indicate a marked decline in the soil’s organic carbon retention capacity relative to its nitrogen content across the elevational gradient (from approximately 30 to 11) (Zhang et al., 2025). The decrease in C/N favors the formation of distinct soil organic matter components, such as particulate organic matter (POM) (average C/N from 16 to 18) and mineral-associated organic matter (MAOM) (from 10 to 12), which possess different stabilities and C/N ratios (Bai and Cotrufo, 2022). The distribution of carbon between MAOM and POM, along with their respective C/N ratios, directly affect soil carbon stocks (Cotrufo et al., 2019). For example, grasslands primarily store more recalcitrant MAOM, which exhibits greater persistence and a higher demand for nitrogen (Cotrufo et al., 2019). Consequently, the observed decrease in C/N with increasing elevational gradient suggests a shift towards a greater proportion of POM and less MAOM at lower elevations, and conversely, less POM and more MAOM at higher elevations (Bai and Cotrufo, 2022).

Furthermore, soil nutrients with varying C/N ratios can selectively enrich specific bacterial groups, such as oligotrophic and copiotrophic taxa (Yao et al., 2017). With increasing altitude, C/N also demonstrates a significant correlation with variations in bacterial functional groups, including chemoheterotrophs and nitrogen-fixing bacteria (Labouyrie et al., 2023). Soil organic matters, largely derived from vegetation, contains compounds that can foster beneficial bacteria by enhancing nutrient acquisition, stress tolerance, and pathogen inhibition (Vives-Peris et al., 2020). For example, organic acids like citric acid and fumaric acid present in root exudates can attract *Bacillus amyloliquefaciens* and *Bacillus subtilis* by promoting their biofilm formation (Zhang et al., 2013). Similarly, plant-derived secondary metabolites, such as flavones, can enrich rhizosphere *Oxalobacteraceae*, thereby improving nitrogen acquisition in maize (Yu et al., 2021). Across increasing altitudes, both interspecific competition and habitat filtering act in concert to shape the soil bacterial community (Zhang et al., 2018). Therefore, a diminished C/N ratio promotes phylogenetic clustering at higher elevations, where stable organic matter and root exudates facilitate deterministic selection, while stochastic processes exert a greater influence on overall community assembly.

At lower elevations, environmental factors did not significantly predict soil bacterial community assembly, whereas at higher elevations, four variables demonstrated a significant influence (Tables 2, 3). Vegetation exerted the strongest influence on phylogenetic conservatism (βNTI), likely due to its capacity to modulate soil physicochemical properties and contribute organic inputs like root exudates and litter, thereby shaping the microbial habitat (Waldrop et al., 2006; Vives-Peris et al., 2020).

### 4.4 Limitations of this study and recommendations for future microbial studies along arid mountain elevational gradients

This study possesses certain limitations that necessitate careful interpretation of the results. Firstly, our sampling was confined to an elevational range (1,707–3,548 m), which may not encompass all landscapes on the northern reaches of the Central Kunlun Mountains, such as the sparsely vegetated alpine scree slopes at 4,200–5,000 m. Secondly, vegetation exhibits numerous features (e.g., diversity, composition, varying carbon and nitrogen content in biomass) that can significantly influence soil bacterial diversity and composition. For future research, comprehensive sampling across the entire elevational gradient, coupled with a detailed investigation of vegetation features, is recommended to further elucidate the diversity, distribution, and underlying ecological processes of soil bacterial communities.

## 5 Conclusion

This study demonstrates that soil bacterial community assembly along arid mountain elevational gradients is primarily governed by the interplay between vegetation and soil stoichiometry, with the soil C/N ratio being a pivotal determinant. While bacterial α-diversity increased with elevation corresponding to enhanced precipitation and vegetation cover, and β-diversity followed a hump-shaped pattern, the overarching driver of community structure was the soil C/N ratio. This highlights a key mechanism where vegetation mediates bacterial assembly by modulating soil nutrient stoichiometry, promoting phylogenetic clustering at higher elevations through stable organic matter inputs. Although deterministic selection strengthens in these resource-modified niches, stochastic processes prevail overall. These findings advance microbial biogeography by identifying soil C/N as a critical factor in arid mountains, providing a framework for predicting ecosystem responses to environmental change.

## Data Availability

The datasets presented in this study can be found in online repositories. The names of the repository/repositories and accession number(s) can be found here: https://www.ncbi.nlm.nih.gov/bioproject/PRJNA1322520/.
