# Supplementary material for: Soil C/N ratio governs bacterial community assembly along an arid mountain elevational gradient
Source: Front Microbiol. 2026 Jan 20;16:1703939. doi: 10.3389/fmicb.2025.1703939 (PMC12864470; doi:10.3389/fmicb.2025.1703939)
Supplement: Supplementary file 1 [file Data_Sheet_1.docx]

**Soil C/N Ratio Governs Bacterial Community Assembly along an Arid Mountain Elevational Gradient**

Yongguang Zhang^1,2†^, Chaonan Li^3†^, Fanjiang Zeng^2^, Bo Zhang^2^, Zhihao Zhang^2^, Chenhong Li^1*^, Hongchen Jiang^4*^

^1^Faculty of Agriculture, Forestry and Food Engineering, Yibin University, Yibin, China

^2^Xinjiang Key Laboratory of Desert Plant Roots Ecology and Vegetation Restoration, Xinjiang Institute of Ecology and Geography, Chinese Academy of Sciences, Urumqi, China

^3^Ecological Security and Protection Key Laboratory of Sichuan Province, Mianyang Normal University, Mianyang, China

^4^School of Life Sciences, Henan University, Kaifeng, China

^†^These authors contributed equally to this work

***Correspondence:**

Li, Chenhong, zixuan_xj@163.com

Jiang Hongchen, ﻿[jiangh@henu.edu.cn](mailto:jiangh@henu.edu.cn)

**Table S1** Geographic information of seven sampling sites

| Site | Elevation (m) | North latitude | East longitude | Landscape |
| --- | --- | --- | --- | --- |
| C1 | 1707 | 36°47′03.5′′ | 80°47′52.5′′ | Gobi Desert (sandy soil covered with sparse plants) |
| C2 | 1960 | 36°36′27.4′′ | 80°44′20.4′′ | Gobi Desert (sandy soil covered with sparce plants) |
| C3 | 2448 | 36°22′57.1′′ | 80°33′53.9′′ | Desert steppe (yellow soil covered with some plants) |
| C4 | 2746 | 36°19′47.6′′ | 80°28′40.3′′ | Desert steppe (sandy soil covered with some plants) |
| C5 | 2905 | 36°15′08.2′′ | 80°20′40.1′′ | Typical grassland (light brown soil covered with many plants) |
| C6 | 3248 | 36°12′13.8′′ | 80°15′45.4′′ | Meadow (deep brown soil covered with many plants) |
| C7 | 3548 | 36°09′52.2′′ | 80°15′14.9′′ | Meadow (deep brown soil covered with many plants) |

| **Table S2** Similarity matrix (Spearman ρ^2^). The function *varclus* in Hmisc R package is used to analyze the collinearity between the different environmental variables. | | | | | | | | | | | | | | |
| --- | --- | --- | --- | --- | --- | --- | --- | --- | --- | --- | --- | --- | --- | --- |
|  | Wat | pH | Con | Sal | TN | AP | NO_3_^-^ | NH_4_^+^ | Veg | C/N | SOC | MAT | MAP | Alt |
| Wat | 1 | 0.58 | 0.72 | 0.73 | 0.58 | 0.1 | 0.03 | 0.22 | 0.79 | 0.63 | 0.6 | 0.93 | 0.93 | 0.93 |
| pH | 0.58 | 1 | 0.72 | 0.74 | 0.32 | 0 | 0.01 | 0.04 | 0.62 | 0.25 | 0.37 | 0.6 | 0.6 | 0.6 |
| Con | 0.72 | 0.72 | 1 | 0.81 | 0.4 | 0 | 0.01 | 0.08 | 0.77 | 0.35 | 0.46 | 0.77 | 0.77 | 0.77 |
| Sal | 0.73 | 0.74 | 0.81 | 1 | 0.33 | 0.01 | 0.01 | 0.09 | 0.65 | 0.27 | 0.41 | 0.76 | 0.76 | 0.76 |
| TN | 0.58 | 0.32 | 0.4 | 0.33 | 1 | 0.21 | 0.22 | 0.41 | 0.7 | 0.62 | 0.92 | 0.59 | 0.59 | 0.59 |
| AP | 0.1 | 0 | 0 | 0.01 | 0.21 | 1 | 0.06 | 0.07 | 0.13 | 0.1 | 0.21 | 0.11 | 0.11 | 0.11 |
| NO_3_^-^ | 0.03 | 0.01 | 0.01 | 0.01 | 0.22 | 0.06 | 1 | 0.48 | 0.01 | 0.22 | 0.14 | 0.01 | 0.01 | 0.01 |
| NH_4_^+^ | 0.22 | 0.04 | 0.08 | 0.09 | 0.41 | 0.07 | 0.48 | 1 | 0.19 | 0.35 | 0.34 | 0.18 | 0.18 | 0.18 |
| Veg | 0.79 | 0.62 | 0.77 | 0.65 | 0.7 | 0.13 | 0.01 | 0.19 | 1 | 0.51 | 0.76 | 0.86 | 0.86 | 0.86 |
| C/N | 0.63 | 0.25 | 0.35 | 0.27 | 0.62 | 0.1 | 0.22 | 0.35 | 0.51 | 1 | 0.41 | 0.51 | 0.51 | 0.51 |
| SOC | 0.6 | 0.37 | 0.46 | 0.41 | 0.92 | 0.21 | 0.14 | 0.34 | 0.76 | 0.41 | 1 | 0.67 | 0.67 | 0.67 |
| MAT | 0.93 | 0.6 | 0.77 | 0.76 | 0.59 | 0.11 | 0.01 | 0.18 | 0.86 | 0.51 | 0.67 | 1 | 1 | 1 |
| MAP | 0.93 | 0.6 | 0.77 | 0.76 | 0.59 | 0.11 | 0.01 | 0.18 | 0.86 | 0.51 | 0.67 | 1 | 1 | 1 |
| Alt | 0.93 | 0.6 | 0.77 | 0.76 | 0.59 | 0.11 | 0.01 | 0.18 | 0.86 | 0.51 | 0.67 | 1 | 1 | 1 |

Abbreviations: Alt, elevational gradient; AP, available phosphorus; C/N, the ratio of soil organic carbon to total nitrogen; Con, electronic conductivity; MAP, mean annual precipitation; MAT, mean annual temperature; NH_4_^+^, ammonium nitrogen; NO_3_^−^, nitrate nitrogen; Sal, total water-soluble salt; SOC, soil organic carbon; TN, total nitrogen; Veg, vegetation cover; Wat, soil moisture content.

**Table S3** Spearman's correlation analysis of the relationships of the environmental variables and α-diversity indexes. The *p*-values are adjusted with the method "fdr".

| Taxa | Environmental variable | Correlation | *p*-values | Adj.*p*-values | Significance |
| --- | --- | --- | --- | --- | --- |
| ACE | pH | 0.6737 | 0.0000 | 0.0000 | *** |
| ACE | Con | -0.6712 | 0.0000 | 0.0000 | *** |
| ACE | Sal | -0.6766 | 0.0000 | 0.0000 | *** |
| ACE | AP | 0.1797 | 0.3016 | 0.3290 |  |
| ACE | NO_3_^-^ | 0.0160 | 0.9277 | 0.9277 |  |
| ACE | NH_4_^+^ | 0.1429 | 0.4115 | 0.4369 |  |
| ACE | Veg | 0.5785 | 0.0003 | 0.0005 | *** |
| ACE | C/N | -0.4846 | 0.0035 | 0.0051 | ** |
| ACE | SOC | 0.4583 | 0.0061 | 0.0085 | ** |
| ACE | MAP | 0.7440 | 0.0000 | 0.0000 | *** |
| Chao1 | pH | 0.6682 | 0.0000 | 0.0000 | *** |
| Chao1 | Con | -0.6593 | 0.0000 | 0.0000 | *** |
| Chao1 | Sal | -0.6670 | 0.0000 | 0.0000 | *** |
| Chao1 | AP | 0.1908 | 0.2723 | 0.3091 |  |
| Chao1 | NO_3_^-^ | 0.0255 | 0.8844 | 0.8951 |  |
| Chao1 | NH_4_^+^ | 0.1434 | 0.4096 | 0.4369 |  |
| Chao1 | Veg | 0.5757 | 0.0003 | 0.0005 | *** |
| Chao1 | C/N | -0.4868 | 0.0034 | 0.0050 | ** |
| Chao1 | SOC | 0.4566 | 0.0063 | 0.0087 | ** |
| Chao1 | MAP | 0.7398 | 0.0000 | 0.0000 | *** |
| Observed | pH | 0.6821 | 0.0000 | 0.0000 | *** |
| Observed | Con | -0.7155 | 0.0000 | 0.0000 | *** |
| Observed | Sal | -0.7266 | 0.0000 | 0.0000 | *** |
| Observed | AP | 0.2080 | 0.2305 | 0.2766 |  |
| Observed | NO_3_^-^ | 0.0983 | 0.5728 | 0.5941 |  |
| Observed | NH_4_^+^ | 0.2485 | 0.1498 | 0.1823 |  |
| Observed | Veg | 0.6337 | 0.0000 | 0.0001 | *** |
| Observed | C/N | -0.5571 | 0.0006 | 0.0010 | ** |
| Observed | SOC | 0.5375 | 0.0010 | 0.0016 | ** |
| Observed | MAP | 0.8119 | 0.0000 | 0.0000 | *** |
| PD | pH | 0.4979 | 0.0023 | 0.0035 | ** |
| PD | Con | -0.5192 | 0.0014 | 0.0022 | ** |
| PD | Sal | -0.5592 | 0.0005 | 0.0008 | *** |
| PD | AP | 0.2052 | 0.2370 | 0.2804 |  |
| PD | NO_3_^-^ | 0.0826 | 0.6358 | 0.6513 |  |
| PD | NH_4_^+^ | 0.1415 | 0.4161 | 0.4369 |  |
| PD | Veg | 0.4583 | 0.0056 | 0.0080 | ** |
| PD | C/N | -0.4387 | 0.0089 | 0.0121 | * |
| PD | SOC | 0.3902 | 0.0212 | 0.0273 | * |
| PD | MAP | 0.6620 | 0.0000 | 0.0000 | *** |
| Shannon | pH | 0.7373 | 0.0000 | 0.0000 | *** |
| Shannon | Con | -0.7775 | 0.0000 | 0.0000 | *** |
| Shannon | Sal | -0.7851 | 0.0000 | 0.0000 | *** |
| Shannon | AP | 0.1981 | 0.2540 | 0.2964 |  |
| Shannon | NO_3_^-^ | 0.1922 | 0.2677 | 0.3080 |  |
| Shannon | NH_4_^+^ | 0.3857 | 0.0228 | 0.0290 | * |
| Shannon | Veg | 0.7186 | 0.0000 | 0.0000 | *** |
| Shannon | C/N | -0.6101 | 0.0001 | 0.0002 | *** |
| Shannon | SOC | 0.6745 | 0.0000 | 0.0000 | *** |
| Shannon | MAP | 0.8515 | 0.0000 | 0.0000 | *** |
| Simpson | pH | 0.7286 | 0.0000 | 0.0000 | *** |
| Simpson | Con | -0.7734 | 0.0000 | 0.0000 | *** |
| Simpson | Sal | -0.7909 | 0.0000 | 0.0000 | *** |
| Simpson | AP | 0.1884 | 0.2784 | 0.3118 |  |
| Simpson | NO_3_^-^ | 0.1793 | 0.3015 | 0.3290 |  |
| Simpson | NH_4_^+^ | 0.3717 | 0.0286 | 0.0358 | * |
| Simpson | Veg | 0.7242 | 0.0000 | 0.0000 | *** |
| Simpson | C/N | -0.6193 | 0.0001 | 0.0002 | *** |
| Simpson | SOC | 0.6728 | 0.0000 | 0.0000 | *** |
| Simpson | MAP | 0.8544 | 0.0000 | 0.0000 | *** |

Abbreviations: AP, available phosphorus; C/N, the ratio of soil organic carbon to total nitrogen; Con, electronic conductivity; MAP, mean annual precipitation; NH_4_^+^, ammonium nitrogen; NO_3_^−^, nitrate nitrogen; Sal, total water-soluble salt; SOC, soil organic carbon; Veg, vegetation cover. Significance level: *P < 0.05, **P < 0.01, ***P <0.001.

**Table S4** The analysis of similarity (ANOSIM) based on Bray-Curtis distances for bacterial communities among elevations. The *p*-values are adjusted with the method "fdr". A greater R-value means a greater difference between two elevations if the *p*-value is less than 0.05.

| Groups | R | *p*-values |
| --- | --- | --- |
| 1707 vs 1960 | -0.004 | 0.437 |
| 1707 vs 2448 | -0.012 | 0.437 |
| 1707 vs 2746 | 0.564 | 0.023 |
| 1707 vs 2905 | 1 | 0.018 |
| 1707 vs 3248 | 1 | 0.018 |
| 1707 vs 3548 | 1 | 0.018 |
| 1960 vs 2448 | 0 | 0.437 |
| 1960 vs 2746 | 0.616 | 0.021 |
| 1960 vs 2905 | 1 | 0.018 |
| 1960 vs 3248 | 1 | 0.018 |
| 1960 vs 3548 | 1 | 0.018 |
| 2448 vs 2746 | 0.584 | 0.027 |
| 2448 vs 2905 | 1 | 0.018 |
| 2448 vs 3248 | 1 | 0.018 |
| 2448 vs 3548 | 1 | 0.018 |
| 2746 vs 2905 | 0.68 | 0.018 |
| 2746 vs 3248 | 0.68 | 0.018 |
| 2746 vs 3548 | 0.952 | 0.018 |
| 2905 vs 3248 | 0.84 | 0.021 |
| 2905 vs 3548 | 1 | 0.018 |
| 3248 vs 3548 | 1 | 0.018 |


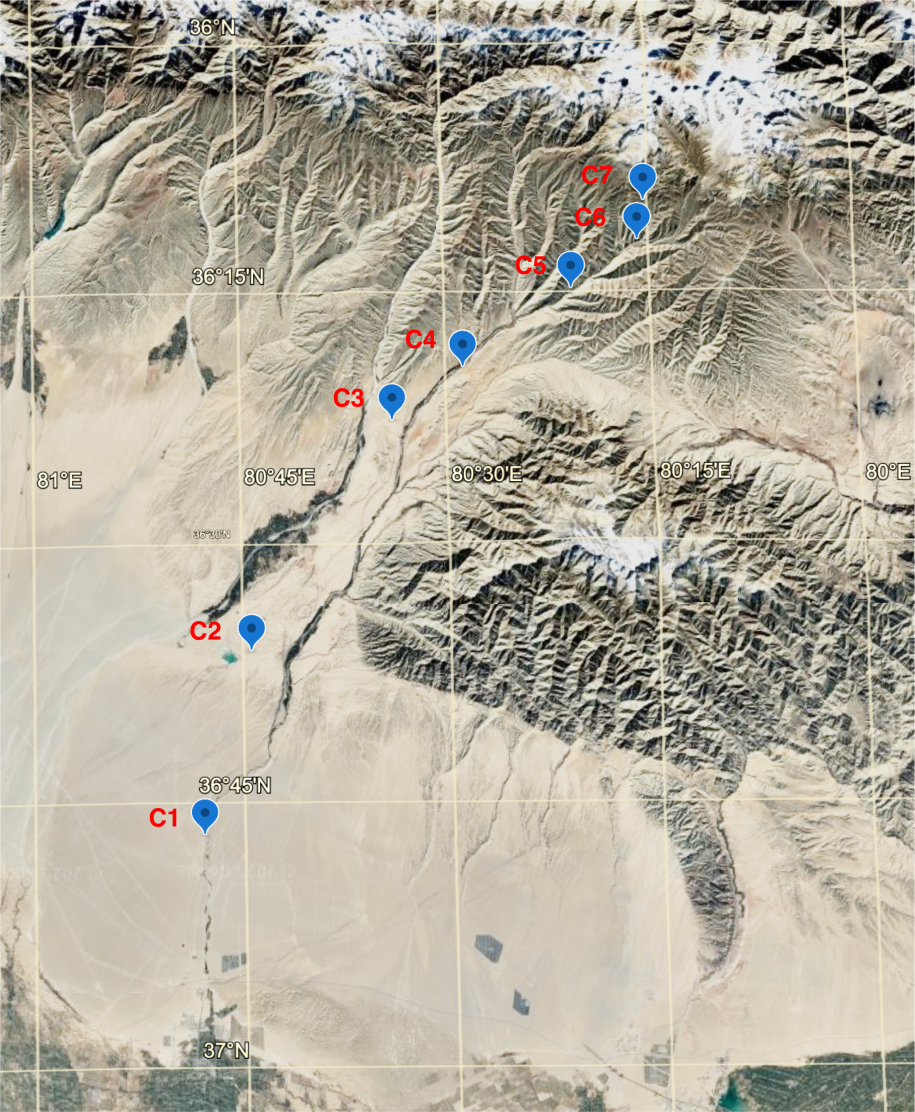


**Fig. S1** Geographic distributions of sampling sites on the northern slope of the Central Kunlum Mountains


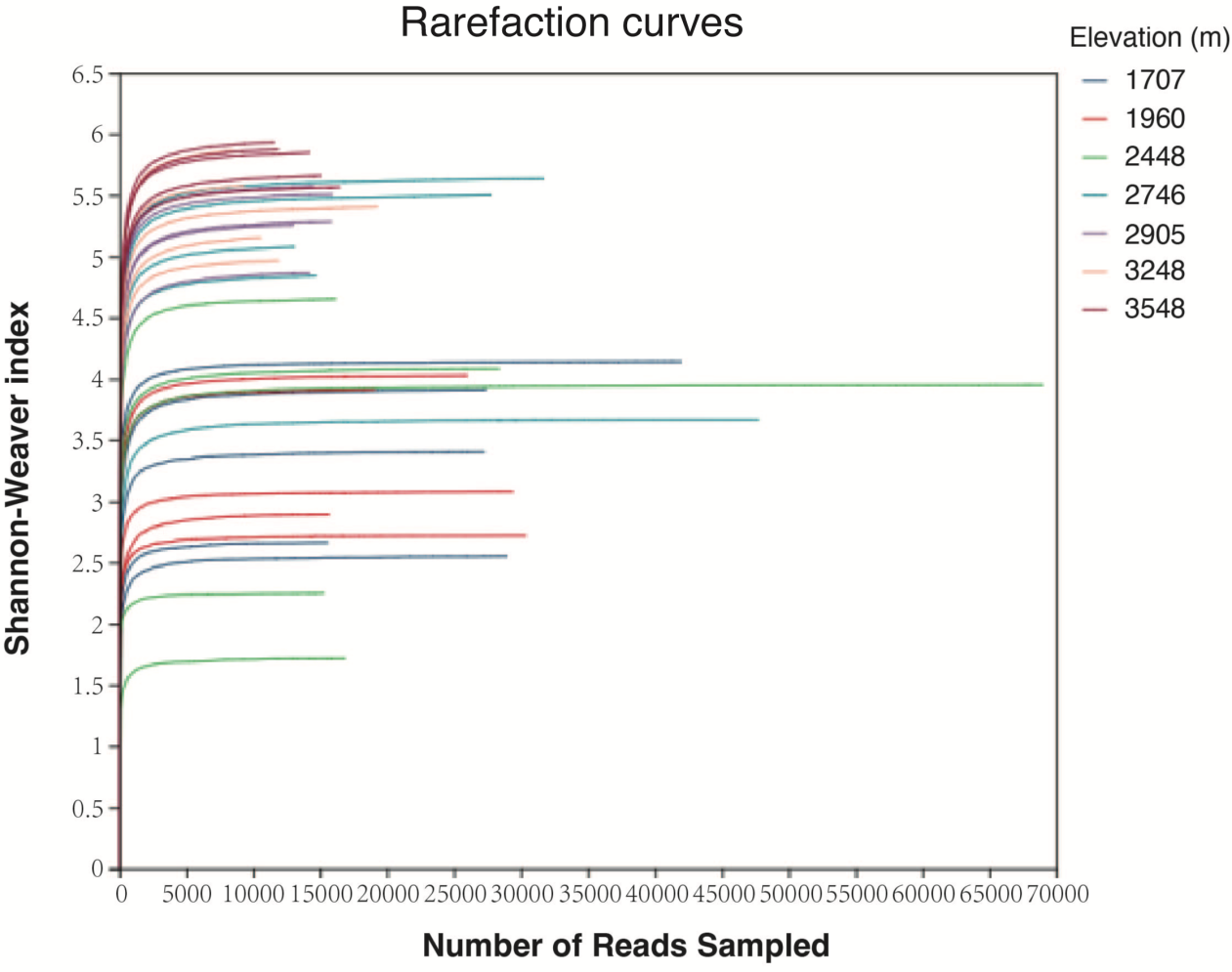


**Fig. S2** Rarefaction curves of Shannon-Weaver index at ASV level


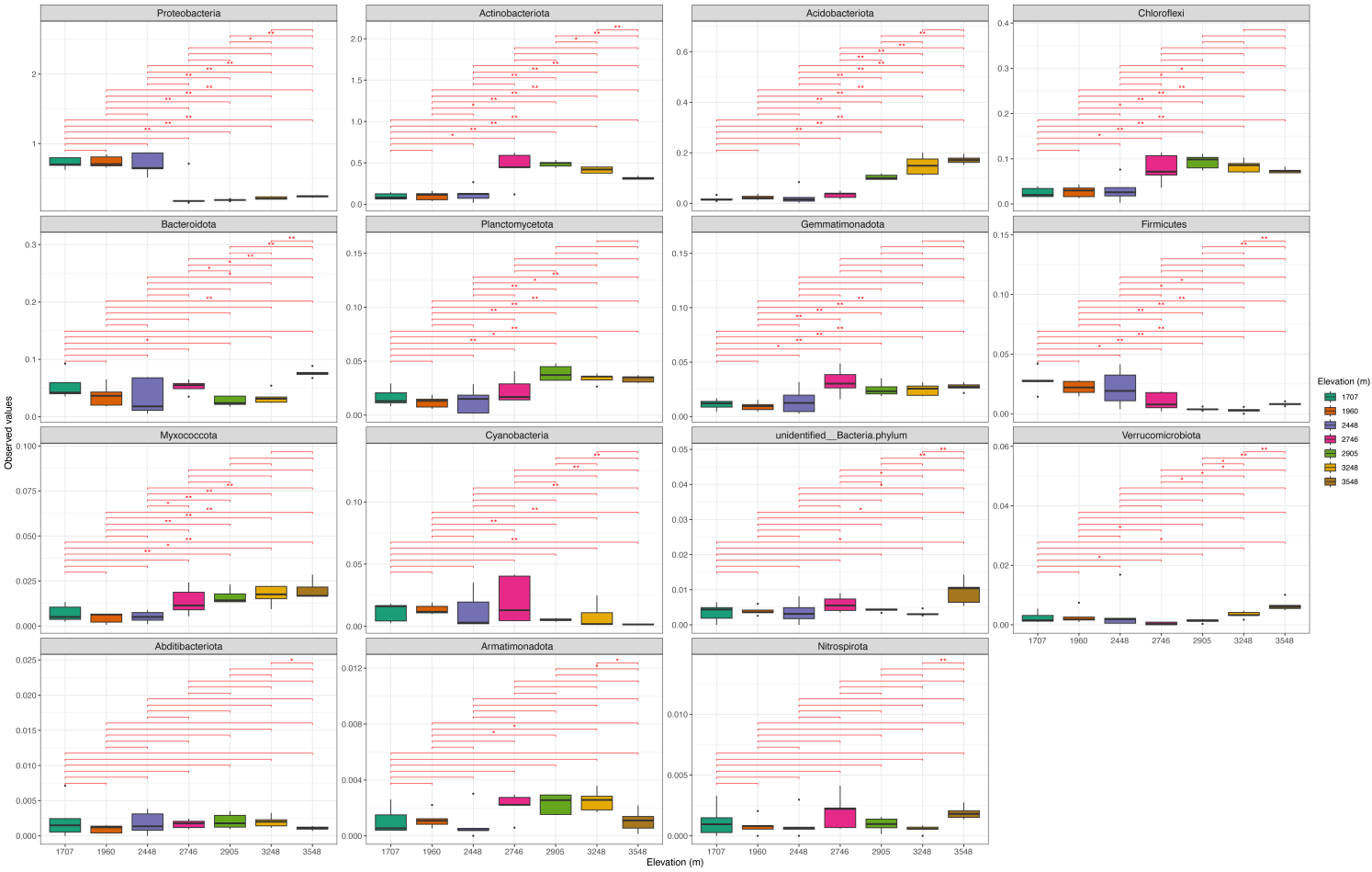


**Fig. S3** The comparisons of relative abundance of top 15 phyla among seven elevational gradients based on the Wilcoxon rank sum test. Significance level: *P < 0.05, **P < 0.01.


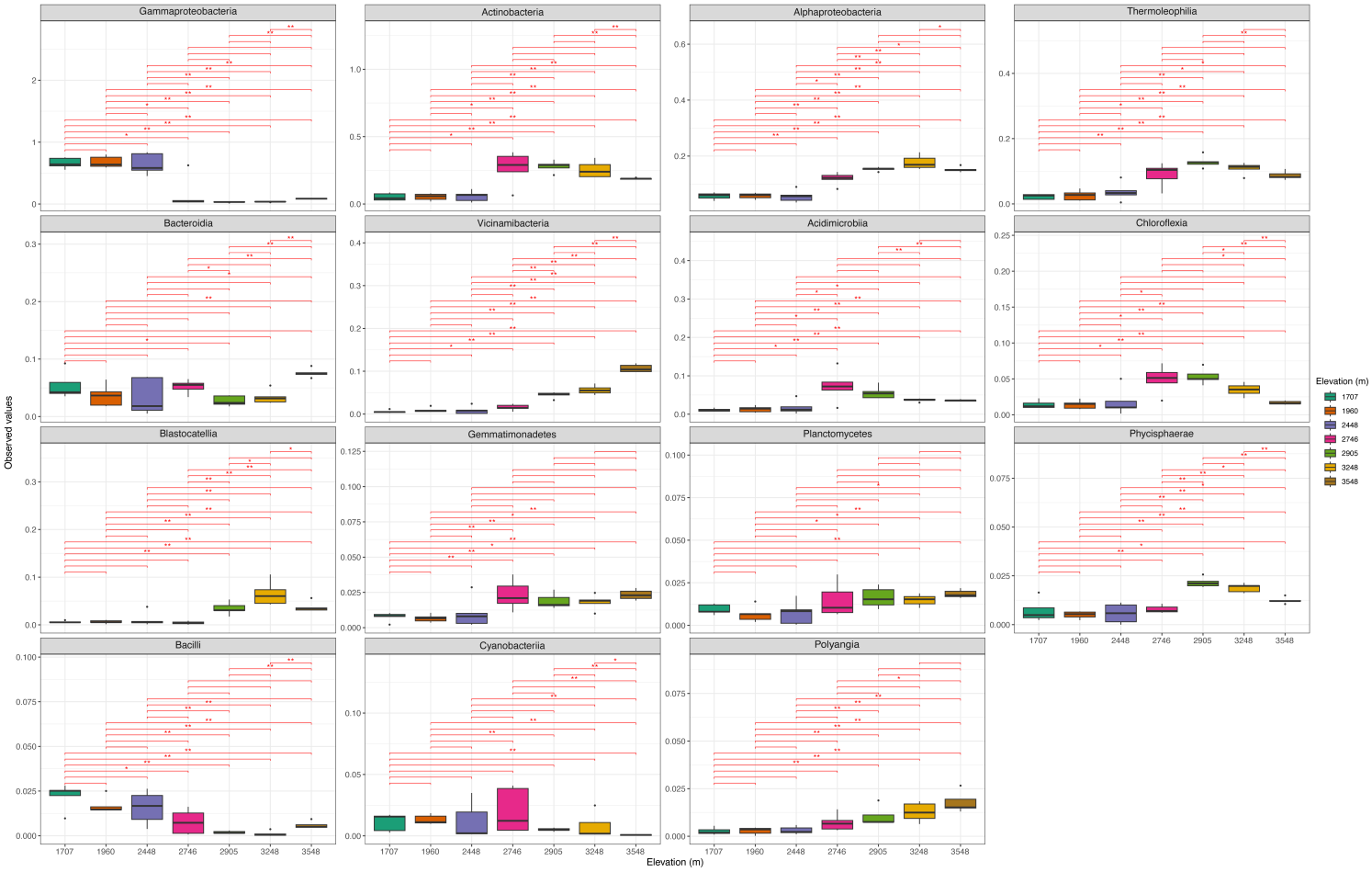


**Fig. S4** The comparisons of the relative abundance in top 15 classes among seven elevational gradients based on the Wilcoxon rank sum test. Significance level: *P < 0.05, **P < 0.01.

**
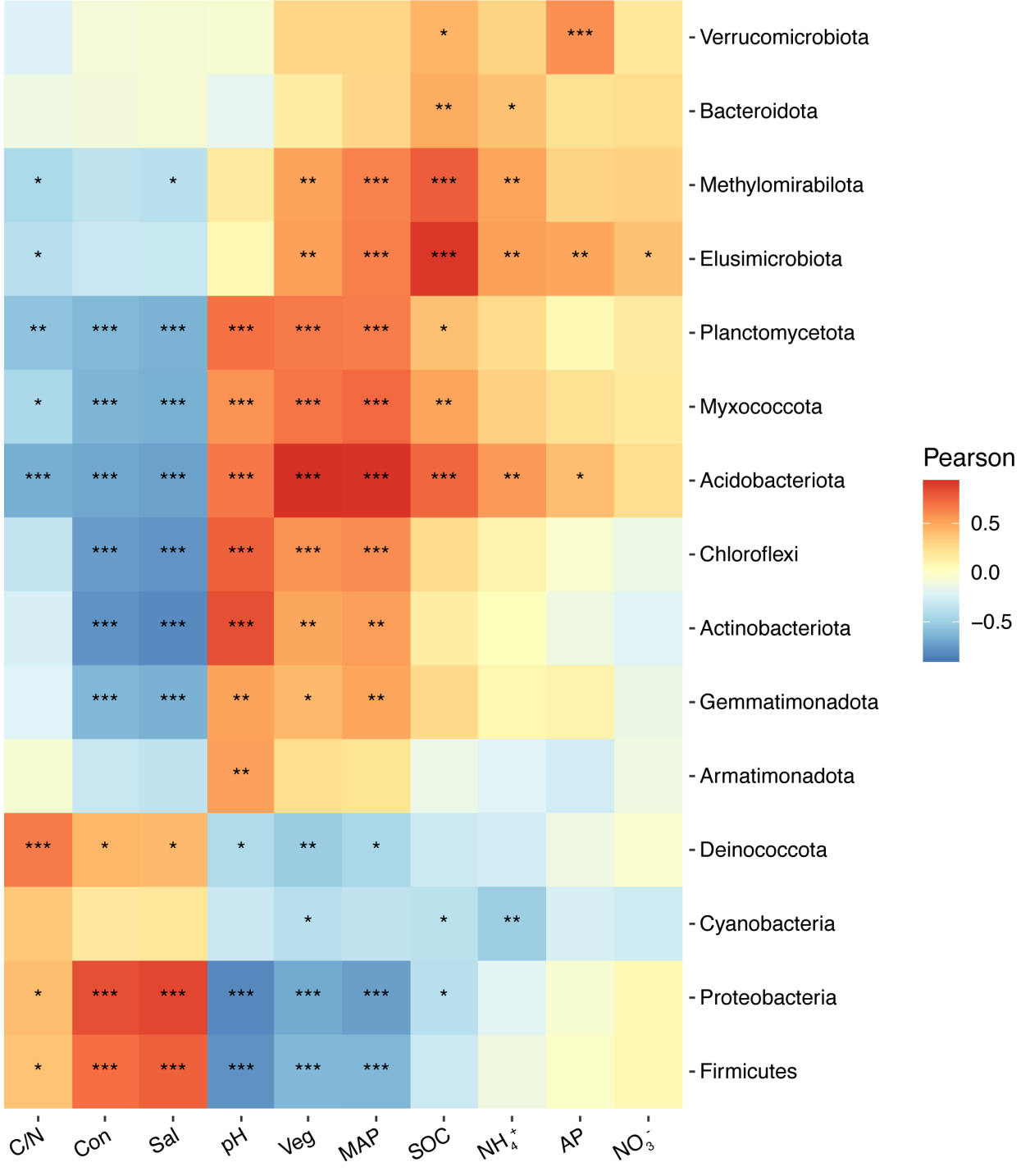
**

**Fig. S5** Spearman correlation between the relative abundance of top 15 phyla and environmental factors. All *p-*values are corrected with the method "fdr". AP, available phosphorus; C/N, the ratio of soil organic carbon to total nitrogen; Con, electronic conductivity; MAP, mean annual precipitation; NH_4_^+^, ammonium nitrogen; NO_3_^−^, nitrate nitrogen; Sal, total water-soluble salt; SOC, soil organic carbon; Veg, vegetation cover. Significance level: *P < 0.05, **P < 0.01, ***P <0.001.

**
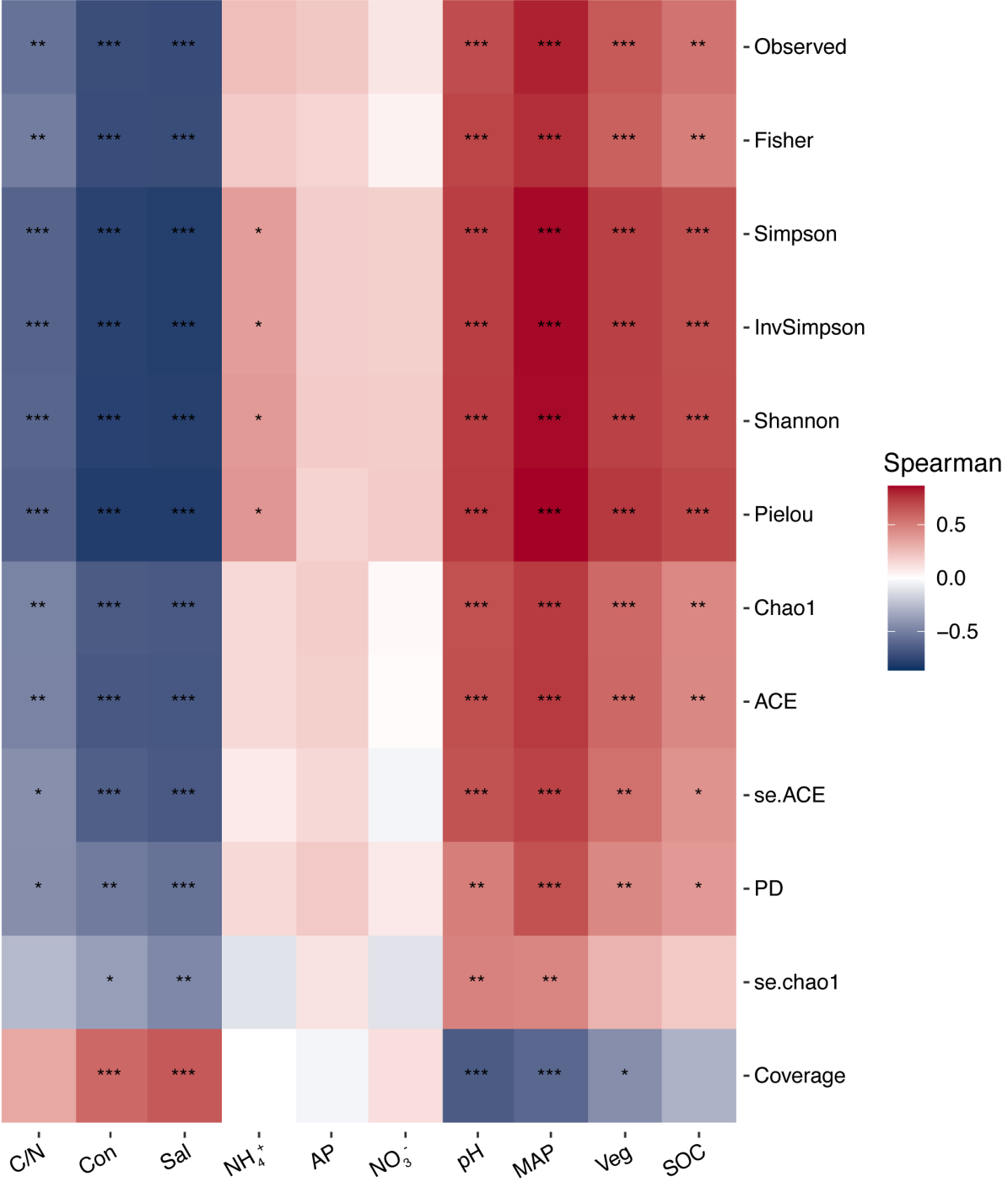
**

**Fig. S6** Spearman correlation between α-diversity indexes and environmental factors. All *p-*values are corrected by the method "fdr". AP, available phosphorus; C/N, the ratio of soil organic carbon to total nitrogen; Con, electronic conductivity; MAP, mean annual precipitation; NH_4_^+^, ammonium nitrogen; NO_3_^−^, nitrate nitrogen; Sal, total water-soluble salt; SOC, soil organic carbon; Veg, vegetation cover. Significance level: *P < 0.05, **P < 0.01, ***P <0.001.

**
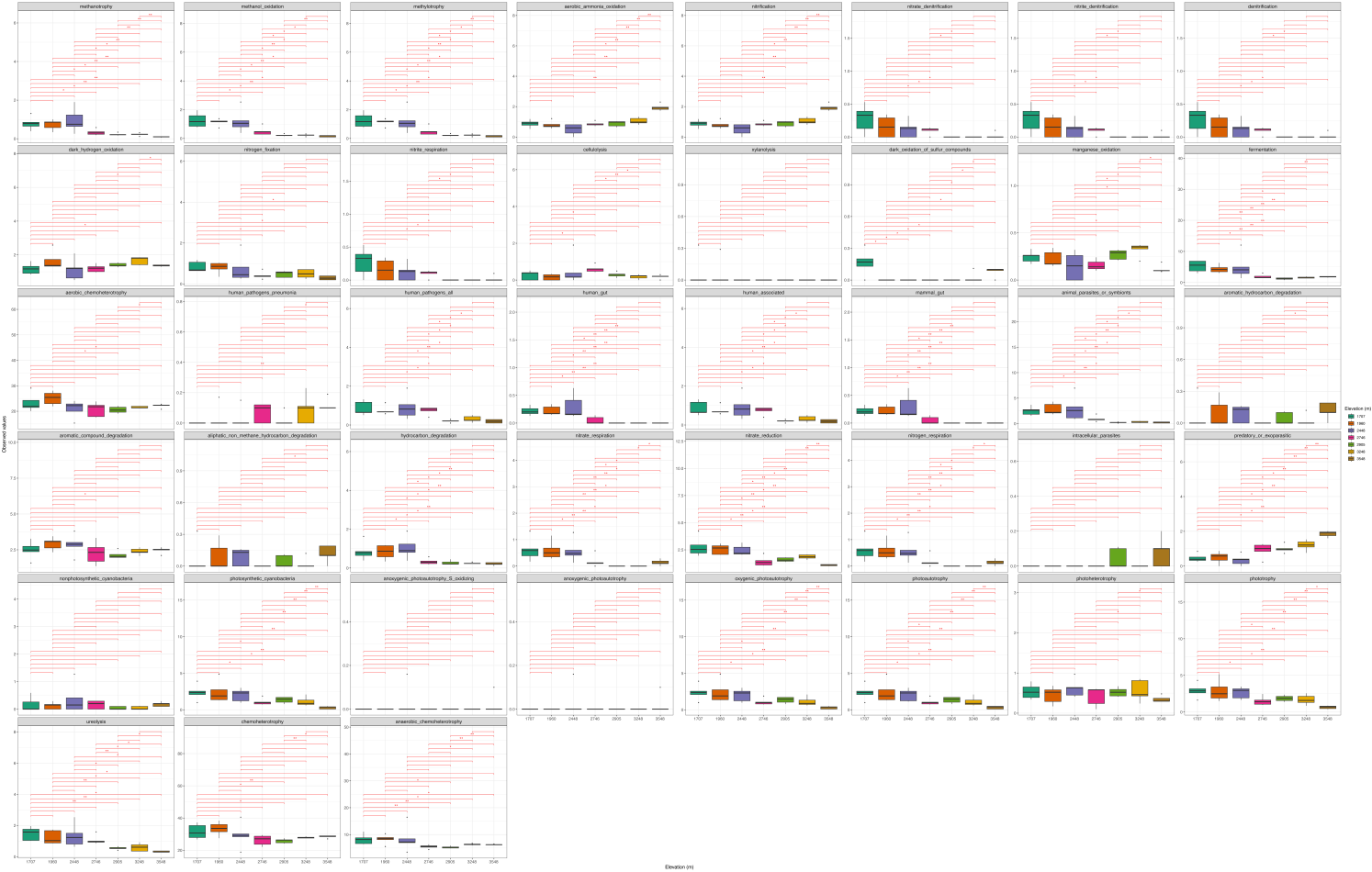
**

**Fig. S7** Shifts of the predicted functional groups along the elevational gradients. The Wilcoxon rank sum test was used to compared the relative abundances of the predicted functional groups, with a false discovery rate (FDR) threshold of <0.005. Significance level: *P < 0.05, **P < 0.01.

**
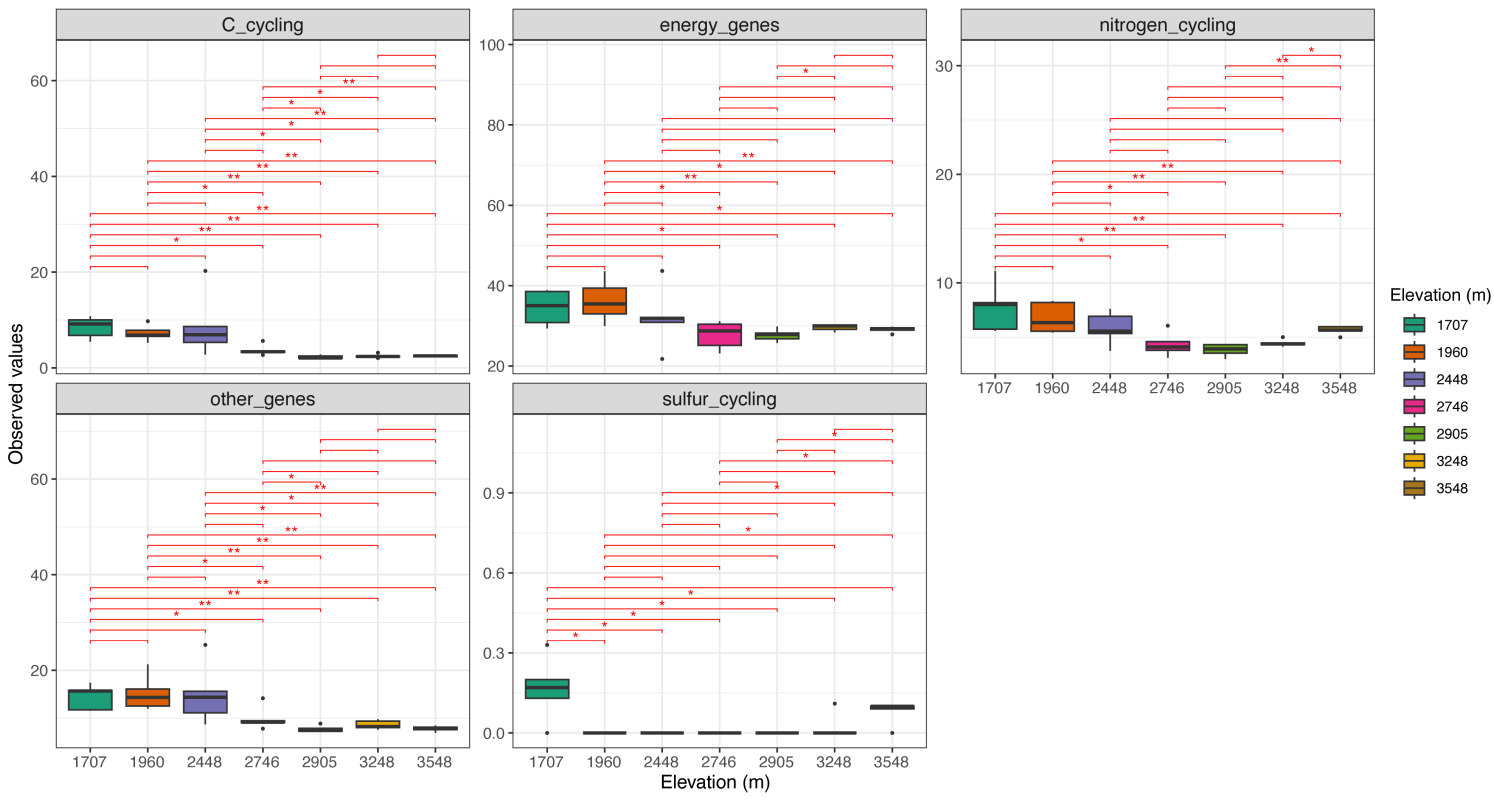
**

**Fig. S8** Shifts of the five types in the predicted functional groups among the seven elevational gradients. The Wilcoxon rank-sum test was used to compared the observed values among the different altitudes, with a false discovery rate (FDR) threshold of <0.005. Significance level: *P < 0.05, **P < 0.01.

**
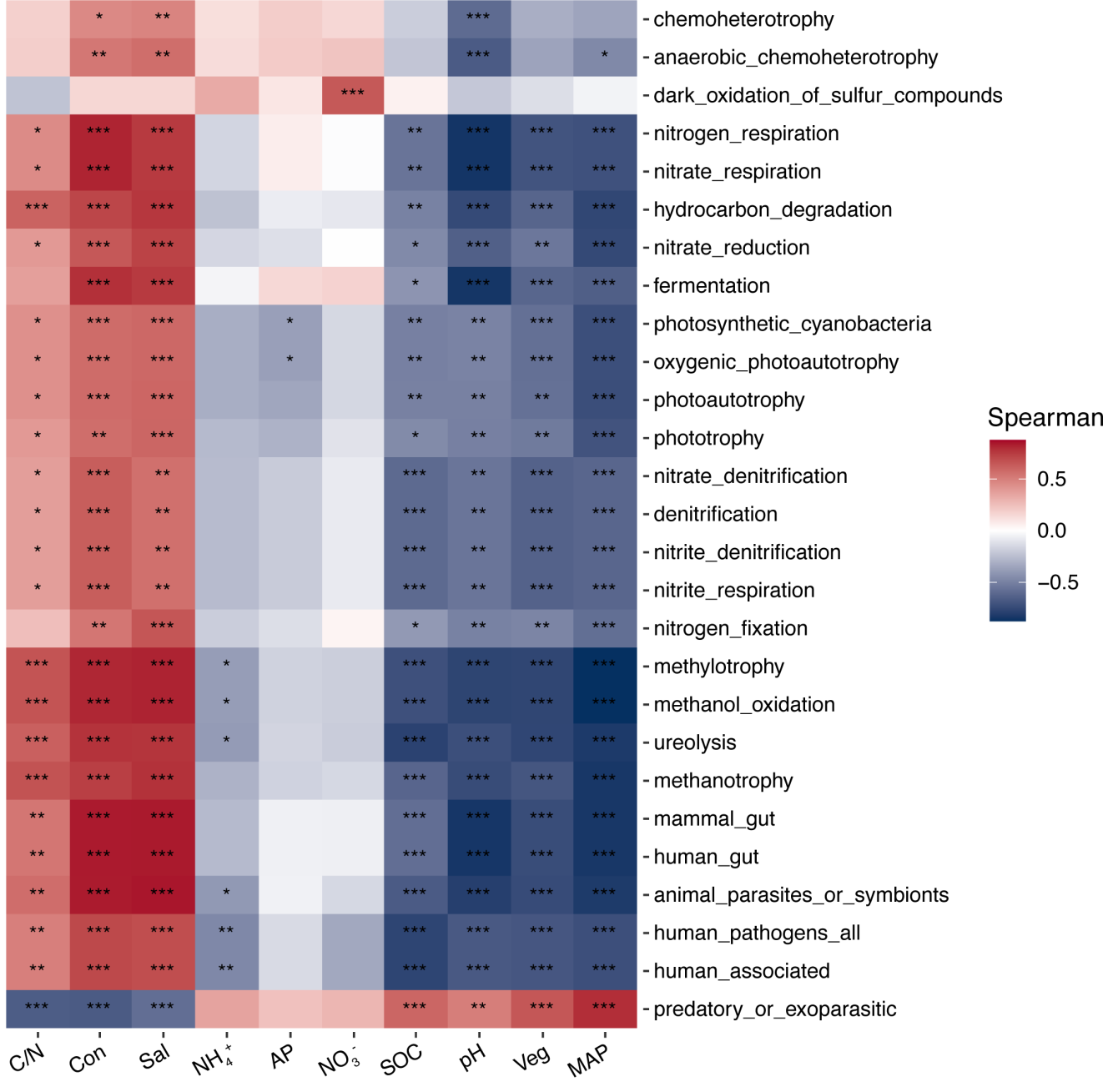
**

**Fig. S9** Spearman correlation between the relative abundance of predicted functional groups and environmental factors. All *p-*values are corrected by the method "fdr". AP, available phosphorus; C/N, the ratio of soil organic carbon to total nitrogen; Con, electronic conductivity; MAP, mean annual precipitation; NH_4_^+^, ammonium nitrogen; NO_3_^−^, nitrate nitrogen; Sal, total water-soluble salt; SOC, soil organic carbon; Veg, vegetation cover. Significance level: *P < 0.05, **P < 0.01, ***P <0.001.
